# Cerebral artery and brain pathology correlates of antemortem cerebral artery 4D flow MRI

**DOI:** 10.1162/imag_a_00322

**Published:** 2024-10-25

**Authors:** Brooke E. Schroeder, Leonardo A. Rivera-Rivera, Madeleine R. Barger, Elena Ruiz de Chavez, Monica Ospina-Romero, Rebecca E. Langhough, Jordan P. Teague, Finnuella J. Carey, Sanjay Asthana, Sterling C. Johnson, Kevin M. Johnson, Laura Eisenmenger, Oliver Wieben, M. Shahriar Salamat, Tobey J. Betthauser

**Affiliations:** Wisconsin Alzheimer’s Disease Research Center, University of Wisconsin School of Medicine and Public Health, Madison, WI, United States; Department of Medicine, University of Wisconsin-Madison School of Medicine and Public Health, Madison, WI, United States; Duke University School of Medicine, Durham, NC, United States; Department of Medical Physics, University of Wisconsin-Madison School of Medicine and Public Health, Madison, WI, United States; Department of Pathology and Laboratory Medicine, University of Wisconsin-Madison School of Medicine and Public Health, Madison, WI, United States; Wisconsin Alzheimer’s Institute, University of Wisconsin School of Medicine and Public Health, Madison, WI, United States; Geriatric Research Education and Clinical Center, William S. Middleton Veterans Hospital, Madison, WI, United States; Department of Radiology, University of Wisconsin-Madison School of Medicine and Public Health, Madison, WI, United States

**Keywords:** cerebrovascular disease, magnetic resonance imaging (MRI), neuropathology, 4D Flow MRI, PCVIPR, hemodynamics

## Abstract

Large-scale clinical research studies often incorporate neuroimaging biomarkers to understand underlying pathologic changes that occur in aging and neurodegenerative disease and are associated with cognitive decline and clinical impairment. Of particular interest are neuroimaging methods designed to understand various aspects of cerebrovascular disease that can lead to dementia and also co-occur with neurodegenerative diseases such as Alzheimer’s disease. Neurovascular 4D flow magnetic resonance imaging is one such method that measures hemodynamic characteristics of medium-large cerebral vessels, but it remains unclear how measures derived from 4D flow imaging including pulsatility index, cerebral blood flow, and cross-sectional area relate to underlying pathologic changes in cerebral arteries and downstream cerebrovascular pathology. For example, pulsatility index is thought to be a marker of vessel compliance, which may be due to fibrotic and/or atherosclerotic changes. This observational study investigates imaging-pathologic correlates of cerebral artery 4D flow MRI in 20 initial brain donors (mean (SD) age at death 78.2 (10.3) years; 3.2 (1.4) years from MRI to autopsy) from the Wisconsin Alzheimer’s Disease Research Center who underwent antemortem imaging and postmortem assessment of cerebral artery and brain pathology to identify possible pathologic correlates of 4D flow MRI. Our results suggest that 4D flow MRI measures recapitulate expected hemodynamic and structural relationships across cerebral arteries, but also that measures like MRI cross-sectional area may reflect arterial fibrosis whereas mean blood flow may indicate downstream cerebrovascular disease, including white matter rarefaction and arteriolosclerosis. In contrast, associations were minimal with pulsatility index and cerebral artery or brain pathology across participants but were moderate across arterial segments. To our knowledge, this is the first study to investigate pathologic correlates of antemortem 4D flow MRI in cerebral arteries. These results provide preliminary insights regarding the pathologic processes contributing to cerebral artery hemodynamics measured with 4D flow MRI that will help inform interpretation of large-scale clinical aging and dementia studies utilizing this method. Future work with larger samples is needed to confirm these findings.

## 1 Introduction

Cerebrovascular disease (CVD) is considered to be a significant contributor to cognitive impairment and dementia in older adults (Gorelick et al., 2011;Jellinger, 2005). Vascular pathology alone is sufficient for the development of cognitive impairment, as evidenced by independent associations of cognitive decline and neurodegeneration with CVD biomarkers (Hachinski et al., 1975;Markesbery, 2001;McVeigh & Passmore, 2006;Román, 2003;Vemuri & Knopman, 2016). Unlike several other dementia-causing diseases, CVD importantly has available and effective primary prevention and treatment (Liu et al., 2021). Vascular brain pathology also commonly co-occurs with other neurodegenerative disease pathology, including Alzheimer’s disease (AD), but the relationships and possible interactions between vascular disease and AD changes remain unclear (Kapasi & Schneider, 2016;Iadecola & Gottesman, 2018;Launer, 2002) with some studies suggesting a causal relationship with AD pathology, others suggesting an interactive effect of CVD and AD on cognitive deficits, and others suggesting age-related parallel processes (Kalaria et al., 2012;Schneider et al., 2007;Vemuri et al., 2015;Zlokovic, 2011).*In vivo*biomarkers for CVD and AD pathology continue to play a key role in understanding the complex relationships between vascular and neurodegenerative disease brain pathology and their impact on cognitive decline and dementia.

Several magnetic resonance imaging (MRI) sequences are available for characterizing different aspects of brain vasculature and CVD, including T2-weighted fluid-attenuation inversion recovery (FLAIR) imaging of white matter hyperintensities attributed to small vessel disease processes, arterial spin labeling (ASL) for tissue perfusion, T_2_*-weighted imagining for microbleeds and hemorrhage, and phase-contrast (PC) imaging to measure vessel morphometry and hemodynamic properties (seeMacDonald & Frayne, 2015for review). Additionally, there are several ongoing efforts to develop and implement new vascular imaging and biofluid protocols designed to comprehensively characterize other aspects of brain vascular pathology (Hinman et al., 2023;Lu et al., 2021;Maillard et al., 2022;Makkinejad et al., 2021;Vemuri et al., 2022). 4D flow MRI is a type of PC MRI that enables characterization of vessel hemodynamics and quantitative assessment of arterial and venous blood flow in large-to-medium size vessels over the full 3D space encompassing brain vasculature (Gu et al., 2005;Johnson et al., 2008). Measures provided from 4D flow include pulsatility index (PI), a presumed measure of vessel compliance as it indicates distal resistance to flow and vascular wall stiffness (Gosling & King, 1974;Roher et al., 2011), mean blood flow in volume per time, and cross-sectional luminal area in mm^2^. Clinical research studies using 4D flow MRI have shown decreased blood flow and increased PI with older age in cerebral arteries, and additionally decreased intracranial blood flow and increased PI in individuals with mild cognitive impairment (MCI) and clinical AD (Rivera-Rivera et al., 2016,^2017^;Roberts, Peret, et al., 2023). While these results highlight the utility of 4D flow MRI to elucidate aging and disease-related vascular changes, the underlying pathological processes impacting 4D flow MRI measures, such as atherosclerosis, are indirectly inferred. Thus, the relationships between pathologic disease and 4D flow metrics remain poorly understood. Furthermore, while several studies have compared antemortem neuroimaging and*ex vivo*MRI (Javierre-Petit et al., 2020;Jonkman et al., 2019;Makkinejad et al., 2021;Nikseresht et al., 2023) outcomes against pathologic characterization for other MR sequences such as T2 FLAIR, volumetric MRI, and susceptibility-weighted imaging (SWI) from T_2_* (Csernansky et al., 2004;Shim et al., 2015;Shoamanesh et al., 2011), we are unaware of any studies that have investigated postmortem correlates of antemortem 4D flow MRI in cerebral arteries. Such information is needed to inform the interpretation of 4D flow MRI findings.

In this work, we investigate pathological correlates of 4D flow imaging in a convenience sample of 20 human brain donors from the Wisconsin Alzheimer’s Disease Research Center (WADRC) who underwent antemortem MR imaging and had postmortem brain tissue and cerebral arteries available for pathological assessment. We investigated the following: 1) age associations for morphology-based cerebral artery pathology and 4D flow measures, 2) local associations between antemortem 4D flow MRI outcomes and cerebral artery pathology in paired arterial segments and secondarily using cerebral artery clusters, and 3) associations between cerebral artery 4D flow MRI and vascular morphometry measures, and neuropathological assessment of brain and cerebrovascular disease assessed according to the 2012 NIA-AA neuropathological examination criteria (Montine et al., 2012) used by the National Alzheimer’s Coordinating Center (NACC).

## 2 Methods

### 2.1 Participants and overall study design

**Fig. 1.**
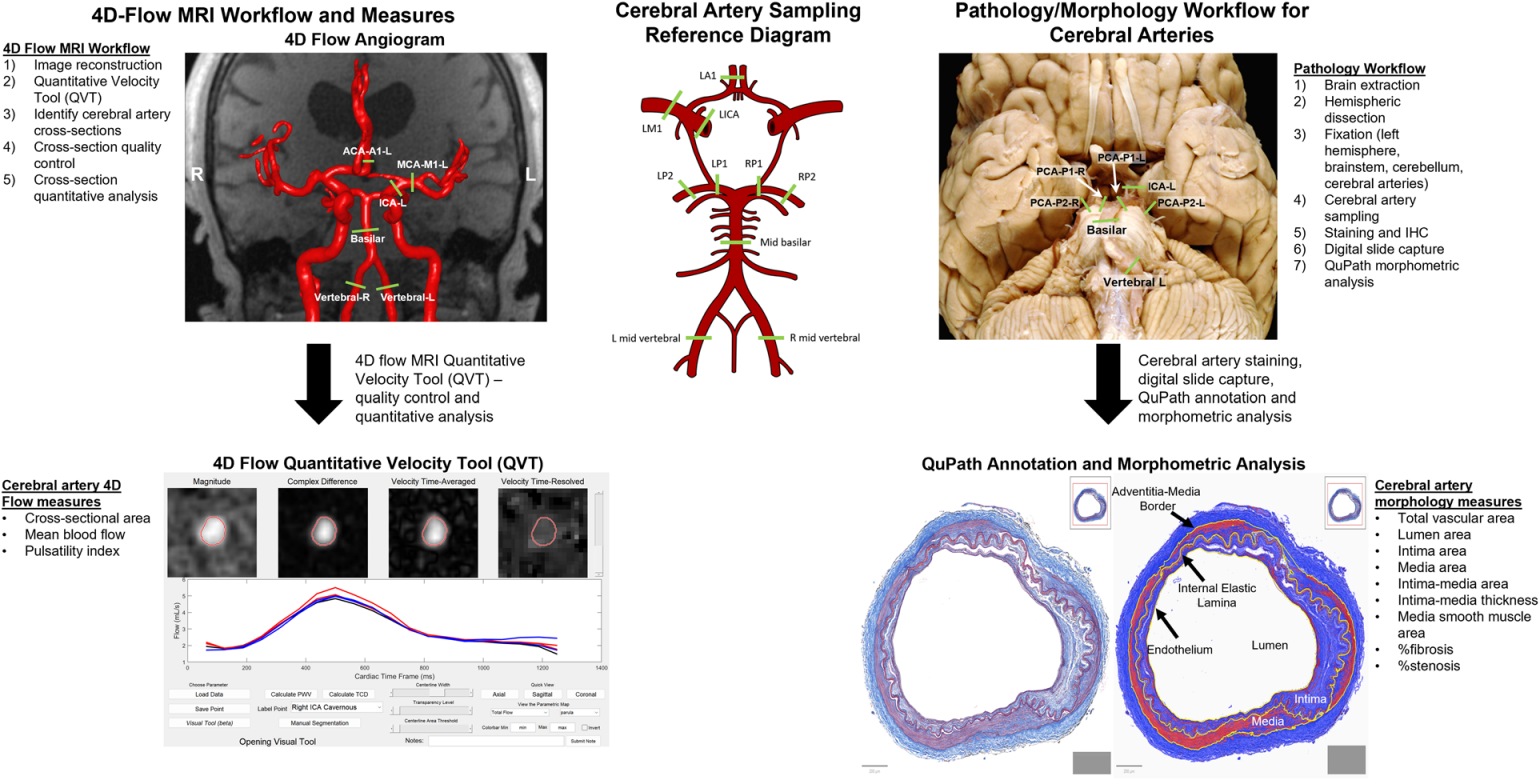
Diagrammatic representation of 4D flow MRI and cerebral artery morphology workflows. Depiction of workflows for generating antemortem 4D flow MRI (left) and postmortem cerebral artery morphology (right) variables for analysis, including a representative angiogram and fixed brain along with the reference diagram (center) used to guide sampling locations for the 10 sampled cerebral artery segments. For ease of visualization, not all cerebral artery cross-sections are labeled in 4D flow angiogram and the fixed brain photo. Additional details for 4D flow MRI and QuPath morphometric processes can be found inSupplemental Figures 2 and 3. The center panel image was adapted fromhttps://commons.wikimedia.org/wiki/File:Circle_of_Willis_unlabeled.svg#filelinks(copyrighted under Creative Commons Attribution-Share Alike 3.0 Unported) and was modified to annotate sampled cerebral artery segments.

Participants from the WADRC were included in the study if they had antemortem 4D flow MRI within 6 years of death (mean (SD) time from MRI to death = 3.2 (1.4) years), were enrolled in and donated brains to the Wisconsin Brain Donor Program (WBDP), and had cerebral arteries collected after death for pathological characterization (n = 20; (mean (SD) age at death = 78.2 (10.3); 11 female; 3 unimpaired, 4 mild cognitive impairment (MCI), 12 dementia, 1 impaired other); seeTable 1for additional details; seeSupplemental Fig. 1for CONSORT diagram; seeFig. 1for pathology and 4D flow MRI workflows). WADRC participants complete a medical history and physical exam, the standard uniform data set (UDS) battery (Weintraub et al., 2009), and structural MR imaging annually or biennially. Included participants were diagnostically characterized in the WADRC’s multidisciplinary consensus conferences using applicable clinical, laboratory, and imaging criteria. Written informed consent was obtained from all study participants prior to imaging and clinical research activities and brain donation. Human studies (antemortem imaging, cognitive testing, health assessments, and questionnaires) were conducted with approval under the University of Wisconsin-Madison Institutional Review Board and in accordance with the Declaration of Helsinki.

**Table 1.** Person demographic information.

	**All participants**
**N**	20
**Female, n (%)**	11 (55.0)
**Age at death, mean (SD) [min, max]**	78.2 (10.3) [57.1, 91.6]
**Age at last 4D flow MRI, mean (SD) [min, max]**	75.1 (10.0) [56.2, 88.7]
**Years from MRI to death, mean (SD) [min, max]**	3.2 (1.4) [0.9, 5.7]
***APOE*** **genotype, n (%)** *	
Homozygous carriers	3 (15.0)
Heterozygous carriers	4 (20.0)
Non-carriers	12 (60.0)
**Last clinical diagnosis, n (%)** **^†^**	
Normal	3 (15.0)
MCI	4 (20.0)
Dementia	12 (60.0)
Impaired—Other	1 (5.0)
**Years from diagnosis to death, mean (SD) [min, max]**	1.0 (1.2) [0, 4.8]
**Clinical diagnosis at MRI, n(%)**	
Normal	4 (20.0)
MCI	5 (25.0)
Dementia	11 (55.0)
Impaired—Other	0 (0.0)

* One participant did not have*APOE*genotype available.

† Impaired other diagnosis was a case with longitudinal decline in executive function that was clinically diagnosed with suspected CTE but later observed to have a glioblastoma upon neuropathological examination.

### 2.2 Antemortem 4D flow MRI acquisition, image processing, and quantification

Whole brain, time-resolved 4D flow MRI data were acquired using 3.0T systems (SIGNA Premier and MR750, GE Healthcare) and a 3D radially undersampled 4D flow MRI sequence (PC VIPR) (Johnson et al., 2008). Data were acquired with the following imaging parameters: Velocity encoding (Venc) = 80 cm/s, imaging volume = 22 × 22 × 10 cm^3^, TR/TE = 7.4/2.7 ms, scan time ≈7 min, acquired spatial resolution = 0.7 mm isotropic, flip angle = 8º, number of projections ≈11,000 (Johnson et al., 2008;Rivera-Rivera et al., 2020). Cardiac triggers were collected for each subject from a photoplethysmogram on a pulse oximeter (GE Healthcare) worn on the subject’s finger during the MRI exam. Cardiac-resolved velocity, magnitude, and angiogram images were retrospectively reconstructed into 20 cardiac phases using 4D flow data and cardiac triggers (Supplemental Fig. 2) (Griswold et al., 2000;Walsh et al., 2000). Magnitude and angiographic images were visually inspected for vessel blurring due to potential participant motion and corrected if necessary (Rivera-Rivera et al., 2022). Images were corrected for background phase offsets and velocity aliasing (Loecher et al., 2016;Roberts, Hoffman, et al., 2023). After image reconstruction and corrections, intracranial arteries including the left anterior (LA1) and middle (LM1) cerebral arteries, left internal carotid arteries (LICA), basilar artery (BA), left and right posterior cerebral arteries (LP1, RP1 and LP2, RP2), and vertebral arteries (LVA, RVA) were segmented automatically in MATLAB (The Mathworks, Natick, MA, United States) using a validated algorithm (https://github.com/uwmri/QVT) that uses a centerline process with local cross-sectional cut-planes automatically placed in every centerline point perpendicular to the axial direction of the vessel (Roberts, Hoffman, et al., 2023;Schrauben et al., 2015). Regions of interest (ROIs) were automatically contoured using a k-means clustering approach under the assumption that any cross-section will contain a low-signal background and a vessel region (Schrauben et al., 2015). Time-averaged cross-sectional area was also estimated based on the magnitude and velocity images from 4D flow. Blood flow rates were estimated from the product of cross-sectional areas and velocities from the automatic segmentation and are reported in mL/cycle. Pulsatility index was calculated using flow measures (PI = (Qmax— Qmin)/Qmean; Q = flow) (Gosling & King, 1974). Cerebral artery segmentation was visually verified for all segments.

**Fig. 2.**
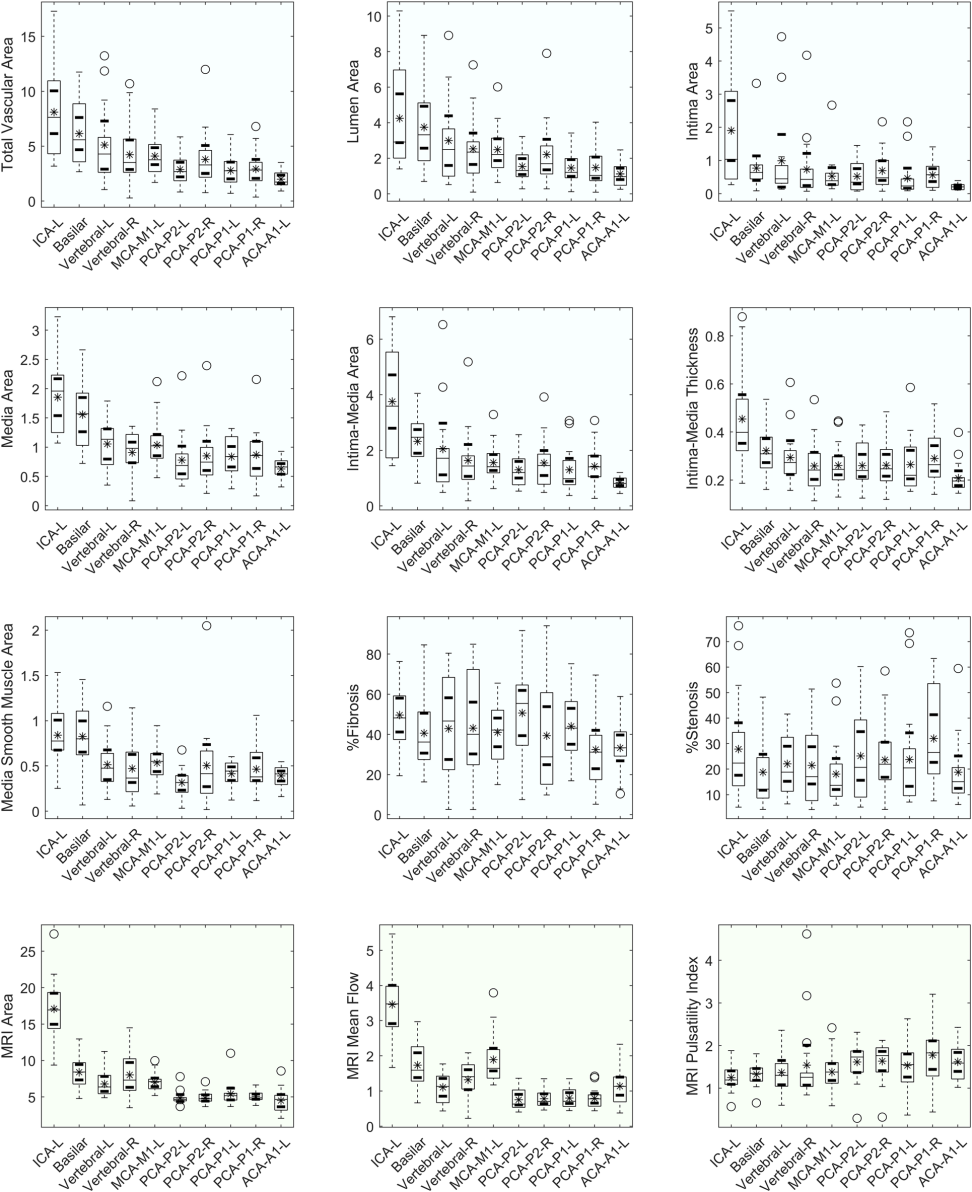
Distributions of cerebral artery morphology and 4D flow MRI measures. Boxplots showing the distributions of all vascular pathology (blue background) and 4D flow MRI (green background) for all cerebral artery segments and participants in the study. Arterial segments are arranged left to right within each boxplot from largest to smallest based on the total vascular area measured from pathology. Boxes indicate the interquartile range with thin lines indicating the median. The mean and 95% confidence interval are represented by an asterisk and thick horizontal lines, respectively. Open circles indicate outliers. ICA = internal carotid artery, MCA = middle cerebral artery, PCA = posterior cerebral artery, ACA = anterior communicating artery. Means and standard deviations for all vascular pathology and 4D flow measures are provided inSupplemental Table 1. All area measures are in mm^2^, intima-media thickness is in mm, mean flow is mL/cycle, and pulsatility index is unitless.

### 2.3 Brain and cerebral artery pathologic assessment

#### 2.3.1 Gross and microscopic brain and vascular pathology assessment

Upon death, whole brain neuropathological examination was performed by a neuropathologist according to NIA-AA guidelines (Montine et al., 2012). After extraction, the brain was weighed, the right hemisphere was manually cut in coronal slabs and frozen, and the left hemisphere, cerebellum, brainstem, and cerebral arteries were fixed in 10% neutral buffered formalin for at least 2 weeks. The formalin fixed brain was manually cut in the coronal plane. Tissue and cerebral arteries underwent gross and microscopic examination, including assessment of brain atrophy and gross evidence of cerebrovascular disease. Braak stage, Thal phase, neuritic plaque scores, “ABC” score, Lewy Body disease (i.e., synucleinopathy), and TDP-43 inclusions were assessed using histochemical and immunohistochemical microscopic evaluation (Montine et al., 2012). Microscopic examination also included qualitative scoring of cerebrovascular disease severity (none, mild, moderate, severe), including cerebral amyloid angiopathy, atherosclerosis, arteriolosclerosis, and white matter rarefaction.

#### 2.3.2 Cerebral artery sampling, microscopic characterization, and morphometry measures

For vascular assessment, LA1, LM1, LICA, basilar artery, LP1, LP2, RP1, RP2, and the left and right vertebral artery segments were sampled, embedded in paraffin, and oriented to produce transverse sections of each vessel. A standard diagram of the cerebral arteries (seeRivera-Rivera et al., 2016) was used to ensure the vessel cross-sections were sampled in similar locations to those used for 4D flow MRI cross-sections (Fig. 1). Vessels were processed into paraffin blocks. Sections were stained with H&E and Masson’s trichome with Verhoeffs elastic stains. Vascular sections were visually assessed for the presence of complete circumferential sections and deeper levels were obtained if needed. Following deeper sectioning, vessels that did not meet quality checks were excluded from the analysis (n = 17/200 vessel segments excluded, seeSupplemental Fig. 1for details).

**Fig. 3.**
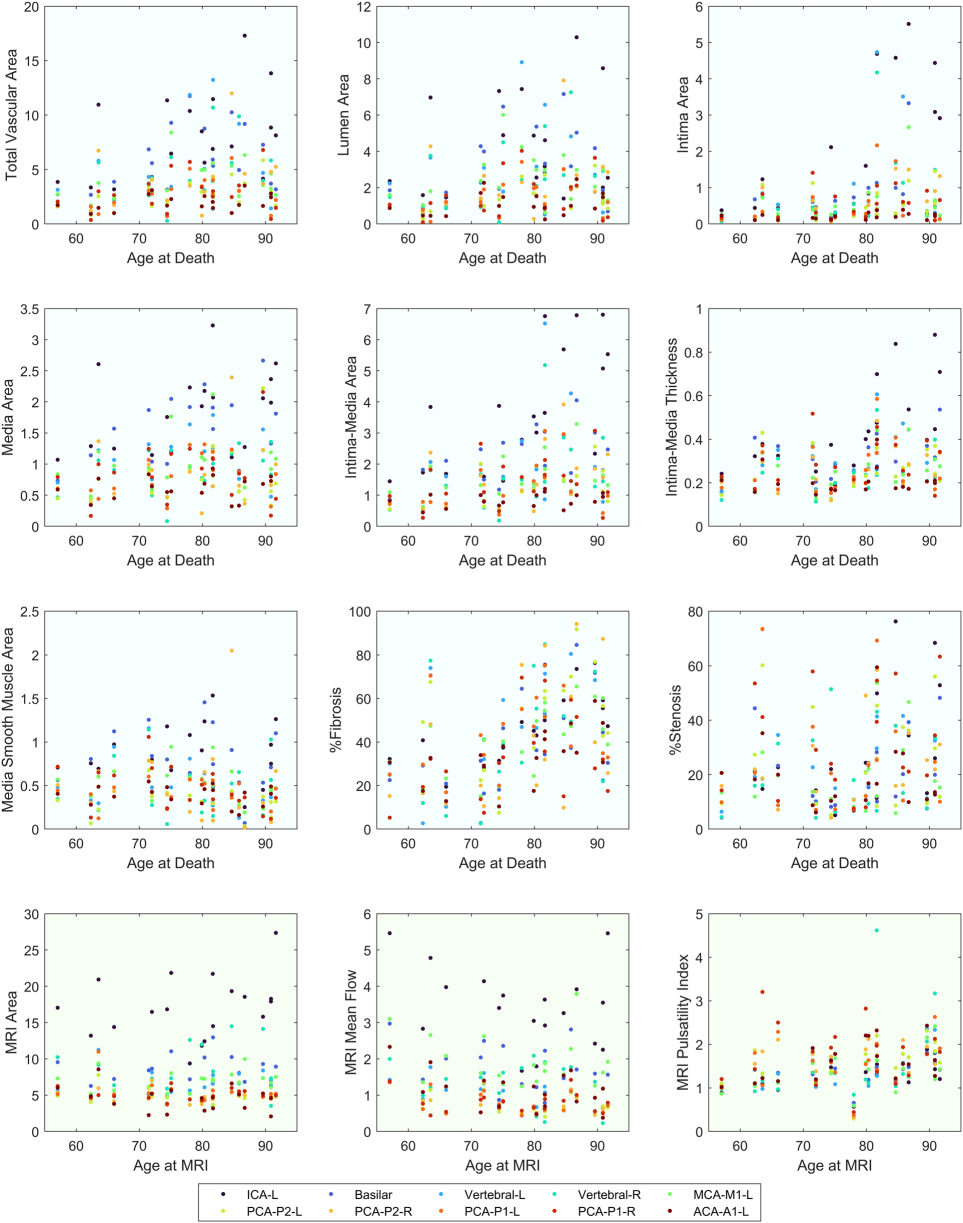
Scatter plots depict the relationship between each vascular morphology (blue background) and 4D flow MRI measure (green background) as a function of age. Datapoints are color coded by cerebral artery segment with vertically aligned observations, indicating observations within a person. Linear mixed-effects model outcomes testing for age associations are provided inTable 3.

Trichrome-stained vascular sections were digitally captured at 20x magnification (0.5039 µm per pixel) using an Aperio AT2 DX digital slide scanner (Leica Biosystems, Nuβlock, Germany). Digital slide images were cropped to include only one vessel per image, and extraneous tissue outside of the vessels was removed using in-house code and MATLAB Software. QuPath software (https://qupath.github.io/version 0.3.0) was used for anatomical annotation and tissue classification (seeSupplemental Fig. 3for an example) to derive morphometry measures of vascular pathology. Using the internal elastic lamina as a defining border between the intima and media, the tunica intima, tunica media, and vascular lumen were manually annotated under the direction of a neuropathologist. For each section, areas representative of collagenous/fibrotic tissue, smooth muscle, and the tunica elastica were manually delineated and used to train a two-way random trees classifier. Following training, the pixel classifier was applied to the entire vascular cross-section to quantify the extent of mural fibrosis and area of smooth muscle cells within the media. Pixel classification was visually quality checked prior to analyses. For all vascular sections, the area (in mm^2^) of the lumen, intima, media, as well as the areas of tissue classified as collagenous/fibrotic tissue and smooth muscle tissue within the media were exported from QuPath. Vascular morphometry measures analyzed included the total vessel area (sum of lumen, intima, and media area), lumen area, intima area (includes plaque when present), media area, the sum of the intima and media area (intima-media area), and the average intima-media thickness (derived from the intima-media area assuming cylindrical geometry), and smooth muscle area in the media. Additionally, we calculated percent stenosis (%stenosis) as the area of expanded intima divided by the area encompassed by the internal elastic lamina (Glagov et al., 1987), and percent fibrosis (%fibrosis) as the percentage area of medial fibrosis divided by the total area of the media.

### 2.4 Statistical analyses

All statistical analyses were conducted using MATLAB 2021a and the Statistics and Machine Learning Toolbox. Analyses were broken into four sections. The first provides descriptive statistics of demographic, clinical, and brain pathology characteristics of the study sample, and characterizes vascular morphometry and 4D flow MRI distributions for all 10 cerebral artery segments. The second characterizes age associations for each of the vascular morphometry and 4D flow MRI outcomes for all 10 cerebral arteries. The third section investigates antemortem-postmortem correlations of paired cerebral artery morphometry and 4D flow MRI outcomes. The last section investigates associations between cerebral artery morphometry and 4D flow MRI, and brain pathology assessed according to the NIA-AA criteria (Montine et al., 2012). Additional details are provided for each section below. Given the small n and exploratory nature of the study, we used an unadjusted significance threshold of α = 0.05 (i.e., results were not corrected for multiple comparisons). For correlational analyses, associations were considered weak if |ρ|<0.3, moderate if between 0.3 and 0.7, and strong for |ρ|>0.7. Subject-level analyses were repeated with time from MRI to autopsy as a covariate. Correlograms were generated using the correlogram function available here:https://www.mathworks.com/matlabcentral/fileexchange/133812-correlogram.

#### 2.4.1 Sample characteristics and outcome variable distributions

Descriptive statistics were generated summarizing key study and sample characteristics and gross neuropathologic assessment. Boxplots were used to depict the distributions of each of the cerebral artery vascular morphometry and 4D flow MRI outcomes by cerebral artery segment. Means and standard deviations for all morphometry and 4D flow MRI outcomes were calculated and reported for each cerebral artery segment.

#### 2.4.2 Age associations with cerebral artery morphology and 4D flow MRI

Since we expect several vascular pathology and 4D flow outcomes to be age-related, we characterized associations between age and each outcome using mixed-effects models to provide context for interpreting the vascular morphometry and 4D flow outcomes. Random artery- and person-level intercepts were used to account for expected within-artery and within-subject correlations. Age at death or age at MRI was the explanatory variable of interest, depending on whether vascular morphometry or 4D flow measures were analyzed, respectively. Age associations were considered significant if the main effect of age had p < 0.05.

#### 2.4.3 Paired cerebral artery vascular morphometry and 4D flow MRI

**Fig. 4.**
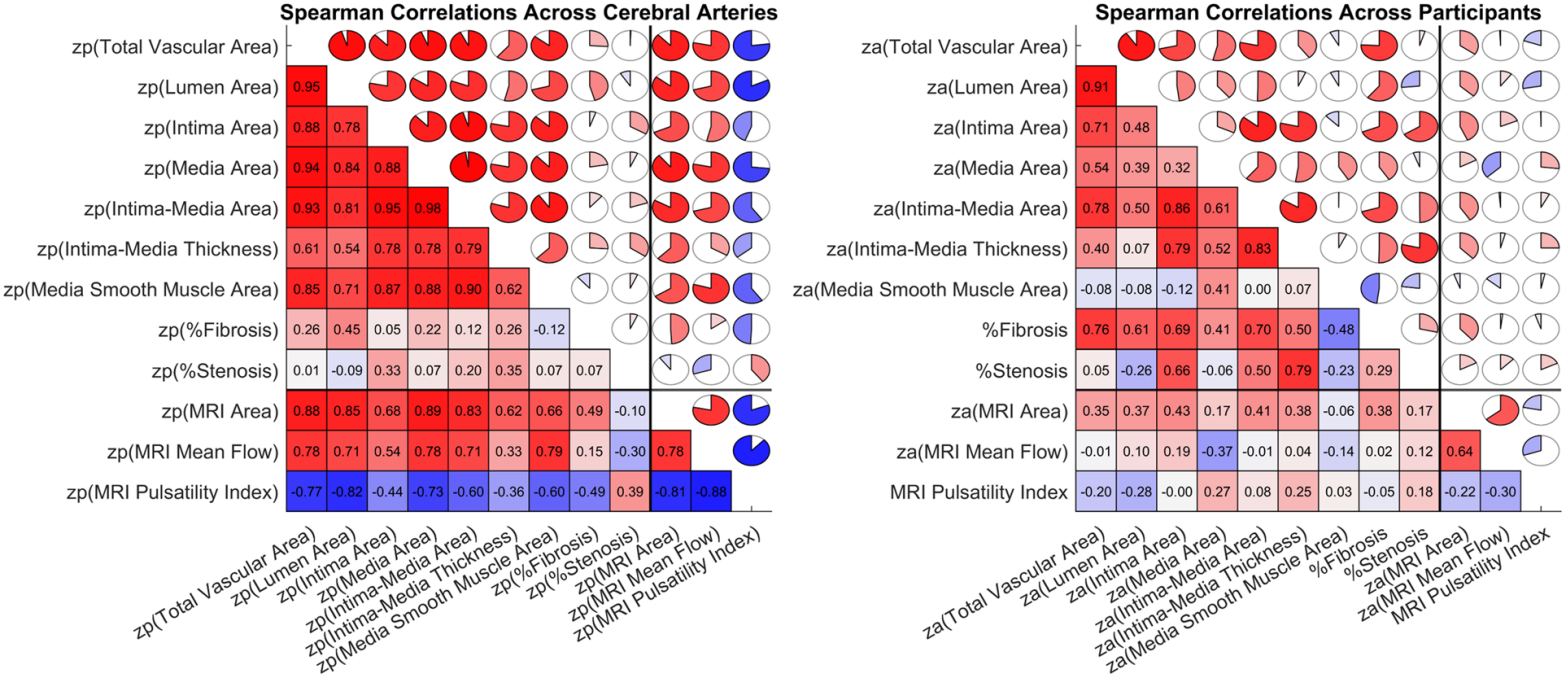
Correlogram of the Spearman correlation coefficients across arteries (left) and across persons (right) for all vascular morphology and 4D flow MRI measures. For artery-level correlations (left), all measures were z-scored at the person level and then averaged across arteries. For person-level correlations (right), measures that indicated artery-level differences by ANOVA were first z-scored using the within-artery means and standard deviations, and then averaged across arterial segments within participants. Correlations between variables were similar when not z-scoring data (data not shown). Red colors indicate positive associations, whereas blue indicates negative correlations. Bold lines differentiate vascular pathology variables (top and left sides) from antemortem MRI measures (bottom and right sides). za indicates measures were z-scored at the artery level. zp indicates measures were z-scored at the person level.

**Fig. 5.**
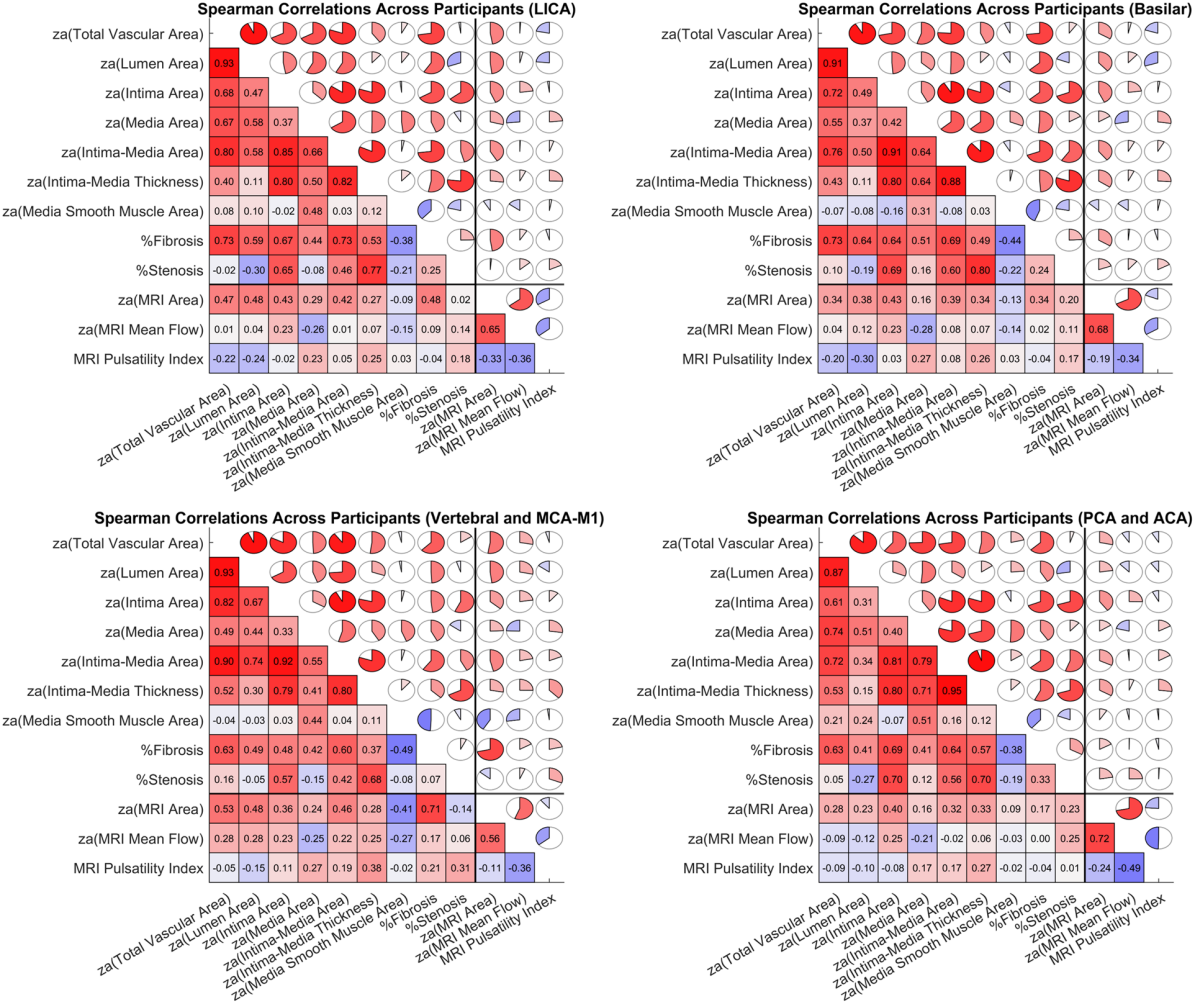
Correlations across subjects for cerebral artery clusters. Artery clusters were determined based on visually apparent clusters of paired-differences between z-scored pathology and MRI measures. za indicates measures were z-scored at the artery level.

Our primary analyses investigated associations between paired cerebral artery morphometry and 4D flow MRI measures (i.e., measures taken from the same arterial segments for antemortem MRI and postmortem arterial pathology) at both the artery-level and person-level. Since we expected outcomes to correlate within-person (e.g., due to chronic vascular changes) and within-artery (e.g., like cerebral arteries will have similar size), outcomes were transformed prior to correlational analyses (seeSupplemental Fig. 4for schematic representation of transformations). A z-score approach, centering values around their mean and dividing by their standard deviation, was used rather than raw values as this allowed us to 1) more easily infer how measures compared across participants relative to others in the sample and across arteries relative to other arteries, 2) combine measures within participants when measures for different arterial segments were missing across participants, and 3) reduce the variance of measures and dimensionality of the comparisons (Andrade, 2021).Artery-level associations: To investigate correlations across arteries, we first determined whether morphology and 4D flow outcomes differed between participants using ANOVA’s. After seeing significant differences between participants for all morphometry and 4D flow MRI outcomes, we z-scored all outcomes using within-person means and standard deviations for each participant individually. These person-level z-scored measures were then averaged across participants for each artery segment resulting in means of person-level z-scored measures (labeled as zp(variable)) for each of the 10 cerebral arteries for each morphometry and 4D flow outcome. Correlograms of Spearman correlations and scatter plots were then used to investigate associations across arteries between cerebral artery morphometry and 4D flow measures.Person-level associations: Similarly, ANOVA’s were used to identify mean differences between arteries for morphology and 4D flow MRI measures. For outcomes indicating mean differences between arteries (all except %fibrosis, %stenosis, and pulsatility index), outcomes were z-scored using within-artery means and standard deviations. These artery-level z-scored measures were then averaged across arteries for each person, resulting in means of artery-level z-scored measures (za(variable)) for each of the 20 participants for each morphometry and 4D flow MRI outcome. Since %fibrosis, %stenosis, and pulsatility index means were not significantly different between arteries, these measures were not z-scored prior to averaging. Correlograms of Spearman correlations and scatter plots were used to investigate associations across participants between cerebral artery morphometry and 4D flow measures. We also performed secondary person-level correlation analyses with artery clusters and for each individual arterial segment since correlations could vary by artery, particularly for arteries with different sizes as suggested by previous studies (Cebral et al., 2008;Gutierrez et al., 2014). The artery clustering approach was used in addition to correlations within individual arteries because we expected this would maintain differences in associations based on artery size and simultaneously would reduce variance, particularly for measurements from smaller arteries that are likely to have poorer signal-to-noise ratios on MRI outcomes. Cerebral artery clusters were identified using the following steps: 1) all morphometry and 4D flow outcomes were z-scored at the artery-level using artery means and standard deviations; 2) ANOVA’s were performed to identify significant paired differences between artery segments for pairs of z-scored morphology and 4D flow measures; and 3) artery clusters were identified based on visual inspection of pairwise comparisons between arterial segments wherein the omnibus test indicated significant paired differences between z-scored morphology and 4D flow measures (seeSupplemental Fig. 5for an example). This resulted in four artery clusters: 1) ICA, 2) mid-basilar artery, 3) the vertebral and MCA-M1 segments, and 4) the PCA and ACA segments.

#### 2.4.4 Associations with NIA-AA pathologic assessment

Lastly, we investigated associations between cerebral artery morphometry and 4D flow MRI measures and variables of interest from neuropathological assessment reported on the NACC form according to NIA-AA criteria (Montine et al., 2012). Within-person means of artery-level z-scored measures (i.e., za(variable)) described in the person-level associations analysis section of 2.4.3 were used for this analysis, except for pulsatility index, %fibrosis, and %stenosis which were averaged without z-scoring as mentioned above. Brain pathology measures included Thal amyloid phase (Thal et al., 2002) (0–5), Braak NFT stage (Braak et al., 2006) (0–6), neuritic plaque score (Mirra et al., 1991) (none, mild, moderate, severe), Lewy body spatial extent (none, limbic, neocortical) (McKeith et al., 2017), TDP-43 (Cairns et al., 2007) presence/absence in the amygdala, TDP-43 presence/absence in the medial temporal lobe (MTL; hippocampus and temporal cortex), and cerebral amyloid angiopathy, atherosclerosis, arteriolosclerosis, and white matter rarefaction severity (none, mild, moderate, severe). We performed ANOVA’s to investigate differences in cerebral artery morphology or 4D flow MRI measures for varying degrees of brain pathology with brain pathology outcomes treated as categorical variables. Secondary analyses were similarly performed using the artery clusters mentioned in the previous paragraph. R^2^, adjusted R^2^, and p-values are reported for all models in the Supplemental Materials and with summarized results presented in the main text. Tukey’s HSD was used to identify pairwise differences when the omnibus test was significant (unadjusted p < 0.05).

## 3 Results

### 3.1 Participant demographic and neuropathologic characterization

Summaries of participant demographic and pathologic characterization are shown inTables 1and2. Most participants were clinically diagnosed with cognitive impairment prior to their death (dementia (n = 12), mild cognitive impairment (MCI; n = 5), unimpaired (n = 3)) and had a mean (SD) 1.0 (1.2) years from diagnosis to death. The mean (SD) time from MRI scan to death was 3.1 (1.5) years. All participants except one had some degree of AD neuropathologic changes, with 15 out of 20 exhibiting intermediate (n = 5) or high (n = 10) AD pathology. Many participants also presented with other neurodegenerative pathology, including Lewy body disease (8/20), medial temporal TDP-43 (16/20), and various indications of vascular pathology (Table 2). Participants demonstrated a range cerebrovascular pathology with mild, moderate, or severe ratings for arteriolosclerosis (17/20), atherosclerosis (17/20), cerebral amyloid angiopathy (13/20), and white matter rarefaction (13/20) upon gross neuropathologic assessment.

**Table 2.** Brain pathology outcomes.

**ABC score, n (%)**	
No AD	1 (5.0)
Low AD	4 (20.0)
Intermediate AD	5 (25.0)
High AD	10 (50.0)
**Arteriolosclerosis, n (%)**	
None	3 (15)
Mild	4 (20)
Moderate	10 (50)
Severe	3 (15)
Atherosclerosis, n (%) **	
None	3 (15)
Mild	10 (50)
Moderate	3 (15)
Severe	3 (15)
Unknown	1 (5)
**Cerebral amyloid angiopathy, n (%)**	
None	7 (35)
Mild	8 (40)
Moderate	1 (5)
Severe	4 (20)
**White matter rarefaction**	
None	7 (35)
Mild	6 (30)
Moderate	6 (30)
Severe	1 (5)
**Old infarcts observed grossly, including lacunes**	5 (25)
**Lewy body disease, n (%)**	
None	12 (60)
Brainstem predominant	0 (0)
Limbic (transitional)	3 (15)
Neocortical (diffuse)	5 (25)
Amygdala predominant	0 (0)
Olfactory bulb	0 (0)
**TDP-43 immunoreactive inclusions**	
Spinal cord	0 (0)
Amygdala	6 (30)
Hippocampus	5 (25)
Entorhinal/inferior temporal cortex	5 (25)
Neocortex	0 (0)
**Frontotemporal lobar degeneration and other tauopathies**	1 (5)

“ABC” score refers to the combination of Thal beta-amyloid phase (A), Braak neurofibrillary tangle stage (B), and CERAD neuritic plaque score (C) according to the NIA-AA guidelines for characterizing Alzheimer’s disease neuropathologic change (Montine et al., 2012).

** One participant did not have atherosclerosis reported.

Distributions of vascular morphometry and 4D flow measures are shown by cerebral artery segment inFigure 2with artery-level means and standard deviations reported inSupplemental Table 1. Morphometry measures followed expected patterns regarding areas of each segment relative to each other (e.g., the ICA had the largest areas). Similarly, MRI area and mean flow generally followed expected patterns regarding size and the expected hemodynamic relationships with higher mean flow in larger arteries with the exception of MCA-M1 mean flow, which was higher than similarly sized arteries.

### 3.2 Cerebral artery morphometry and 4D flow MRI associations with age

Morphometry and 4D flow MRI measures for all cerebral artery segments and participants are shown inFigure 3as a function of age.Table 3summarizes age fixed effects statistics from linear mixed-effects models (random person and artery intercepts). Significant positive age associations were observed with postmortem vascular area, intima area, media-intima area and thickness, %fibrosis, and 4D flow pulsatility index. Age associations were not significant for postmortem lumen area, media area, media smooth muscle area, %stenosis, 4D flow area, and mean flow.

**Table 3.** Cerebral artery morphology and 4D flow MRI age associations.

**Pathology/MRI**	**Variable**	** β _age_**	** 95% CI(β _age_ ) **	** p _age_**
Pathology	vascular area	0.072	0.014, 0.130	**0.015**
Pathology	lumen area	0.033	-0.011, 0.076	0.14
Pathology	intima area	0.028	0.006, 0.050	**0.014**
Pathology	media area	0.010	-0.002, 0.023	0.09 ^†^
Pathology	media-intima area	0.039	0.015, 0.062	**0.0013**
Pathology	media-intima thickness	0.0037	0.0007, 0.0067	**0.016**
Pathology	media smooth muscle area	-0.003	-0.011, 0.004	0.37
Pathology	%fibrosis	0.87	0.27, 1.46	**0.005**
Pathology	%stenosis	0.29	-0.14, 0.71	0.19
MRI	area	0.008	-0.045, 0.061	0.77
MRI	mean flow	-0.016	-0.032, 0.001	0.070 ^†^
MRI	pulsatility index	0.016	0.001, 0.031	**0.038**

Mixed-effects models with artery-level and person-level random intercepts were used to test for associations of variables with age (outcome ~ age + (artery|1) + (person|1)). The analysis includes data from all 20 participants and 10 cerebral artery segments. Age at death was used for vascular pathology measures, whereas age at MRI was used for 4D flow MRI measures. Boldface indicates unadjusted p < 0.05.^†^Indicates unadjusted 0.05 ≤ p< 0.1.

### 3.3 Associations between paired cerebral artery morphometry and 4D flow MRI

**Fig. 6.**
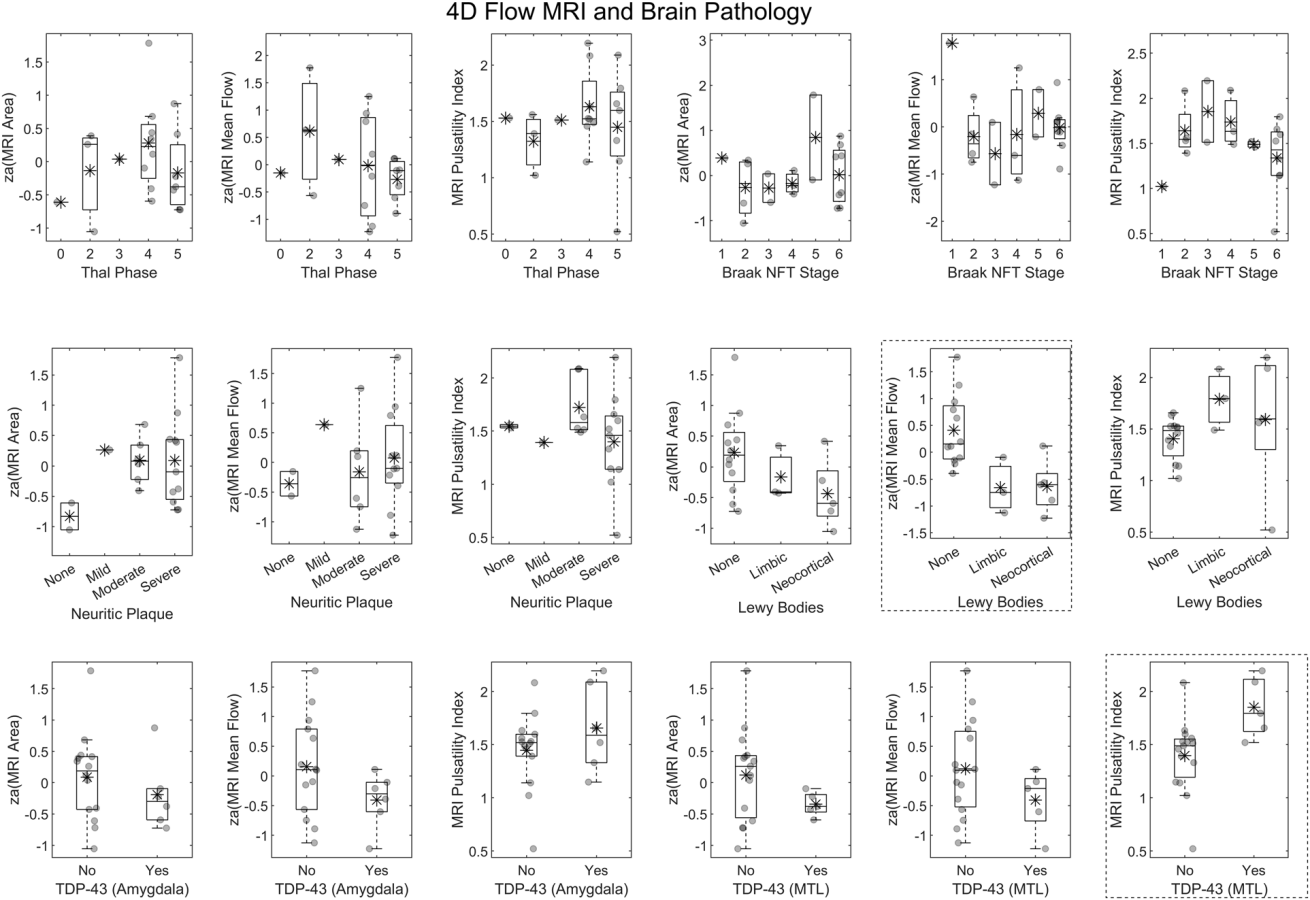
Boxplots showing 4D flow MRI outcomes across neuropathological examination outcomes. Boxes enclosed with dashed lines indicate p < 0.05. R^2^, adjusted R^2^, and p-values for all ANOVAs are provided inSupplemental Table 2. HC = hippocampus; za indicates measures were z-scored at the artery level and averaged within participants.

**Fig. 7.**
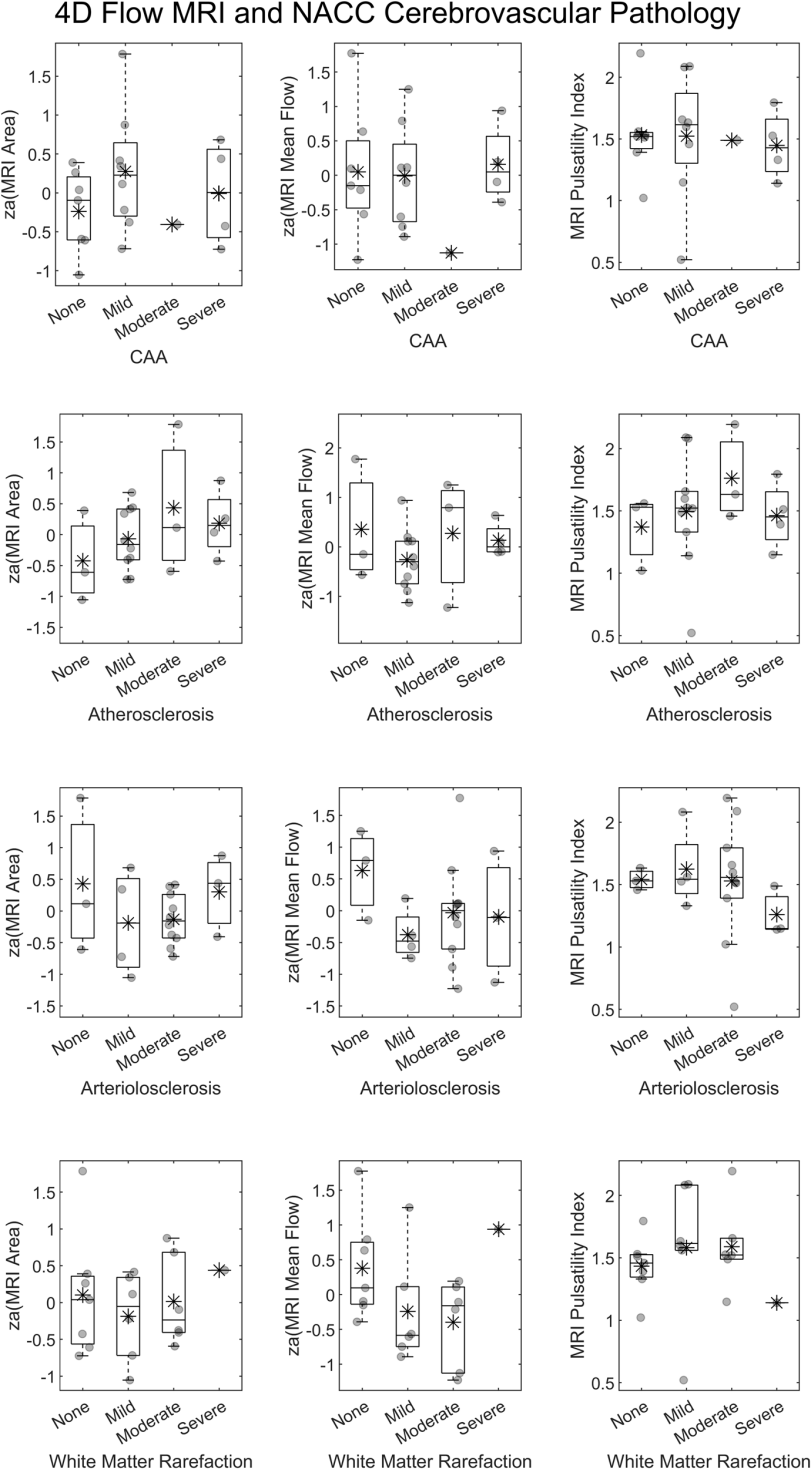
Distributions of 4D flow MRI values for cerebrovascular pathology outcomes reported on NACC neuropathological examination. za indicates measures were z-scored at the artery level and averaged within participant.

Correlograms of morphometry and 4D flow measures are shown inFigure 4with correlograms for artery clusters shown inFigure 5and individual artery correlograms shown inSupplemental Figure 6. Artery-level and person-level scatter plots of all outcome pairs are shown inSupplemental Figures 7 and 8, with person-level scatter plots for artery clusters shown inSupplemental Figure 9. Results in this section are presented in three subsections (vascular morphometry intercorrelations, 4D flow MRI intercorrelations, and correlations between vascular morphometry and 4D Flow MRI) to simplify presentation.

#### 3.3.1 Vascular morphometry intercorrelations

Spearman correlations of morphometry measures across arteries were generally moderate to high (0.54 to 0.95) for most area and thickness measures with weaker associations observed between these measures and %fibrosis and %stenosis (0.01 to 0.45). Correlations of vascular pathology measures across participants ranged from none (0.00) to strong (0.91). Generally, associations with %fibrosis and %stenosis and other morphometry measures were stronger taken across participants with absolute value of correlations ranging from 0.41 to 0.76. Strongest positive correlations with %fibrosis were observed with artery total area (0.76), intima-media area (0.70), intima area (0.69), and lumen area (0.61), with moderate positive associations observed between %fibrosis and intima-media thickness (0.50) and media area (0.41), and a moderate negative association was observed with media smooth muscle area (-0.48). High to moderate positive associations across participants were also observed between %stenosis and intima-media thickness (0.79), intima area (0.66), and intima-media area (0.50).

#### 3.3.2 MRI intercorrelations

Correlations between MRI area, mean flow, and pulsatility index across arteries were strong with mean flow positively correlated with MRI area (0.78) and strong negative correlations between pulsatility index and MRI area (-0.81) and mean flow (-0.88). 4D flow MRI intercorrelations across participants were weaker with a moderate positive correlation observed for mean flow and MRI area, and weak negative associations between pulsatility index and MRI area (-0.22) and mean flow (-0.30).

#### 3.3.3 MRI to vascular morphometry correlations

Correlations between 4D flow MRI and vascular morphometry measures across arteries were generally moderate to high (|rho| range 0.44–0.89) with the exception of correlations with %stenosis (-0.10, -0.30, and 0.39 for MRI area, mean flow, and pulsatility index, respectively), and between intima-media thickness and mean flow (0.33) and pulsatility index (-0.36), and %fibrosis and mean flow (0.15). Correlations between vascular morphometry and 4D flow across person were generally none to moderate with the highest associations observed between MRI area and intima area (0.43), intima-media area (0.41), intima-media thickness (0.38), %fibrosis (0.38), lumen area (0.37), and total vascular area (0.35), and between mean flow and media area (-0.37). Across-person correlations between pulsatility index and vascular pathology measures were none to weak (|rho| ≤0.28). Across-person correlations were similar when accounting for the partial correlation of time from MRI to autopsy (data not shown).

Across-person correlation patterns were similar for artery clusters (Fig. 5) but with higher pathology to MRI correlations observed in the vertebral artery and MCA-M1 cluster, lower for the ICA and for the basilar artery, and weakest for the PCA and ACA cluster (|rho| ≤0.40). Notably, moderate to strong associations were observed in the vertebral and MCA cluster between MRI area and %fibrosis (0.71), total vascular area (0.53), lumen area (0.48), intima-media area (0.46), media smooth muscle area (-0.41), and intima area (0.36), and between pulsatility index and intima-media thickness (0.38) and %stenosis (0.31).

### 3.4 Brain pathology and cerebral artery morphology and 4D flow MRI

Boxplots of 4D flow MRI outcomes are shown for NACC brain pathology and cerebrovascular outcomes inFigures 6and7with ANOVA results provided inSupplemental Table 2. Pulsatility index, mean flow, and 4D flow lumen area did not differ by Thal amyloid phase, Braak NFT stage, neuritic plaque score, presence of amygdalar TDP-43, cerebral amyloid angiopathy severity, atherosclerosis severity, arteriolosclerosis severity, nor white matter rarefaction severity (p > 0.09). Mean flow differed with Lewy body pathology (p = 0.015) such that those with no Lewy body pathology had higher mean flow compared to those with limbic Lewy body pathology. Pulsatility index and MRI area did not differ by Lewy body pathology (p > 0.55). Higher pulsatility index was observed for those with medial temporal TDP-43 pathology (p = 0.017), but no differences were observed for mean flow or MRI area. Although not reaching significance, MRI area and pulsatility index were generally higher with increasing severity of atherosclerosis (p = 0.44 and 0.66, respectively), and mean flow was lower for those with mild, moderate, or severe arteriolosclerosis compared to those with no arteriolosclerosis (p = 0.43). Mean flow was also lower for those with mild-moderate white matter rarefaction compared to those with no white matter rarefaction, but this was not significant (p = 0.16). Results did not differ when including time from MRI to autopsy as a covariate (data not shown).

Boxplots of arterial morphology outcomes for NACC brain pathology and cerebrovascular outcomes are provided inSupplemental Figures 10, 11, and 12with ANOVA results provided inSupplemental Table 2. Generally, arterial pathology was not different by Thal phase, Braak Stage, Lewy body extent, nor TDP-43 presence/absence except for one person with high intima-media thickness and Thal phase 3 that differed from other Thal phases. Several vascular morphology outcomes differed by neuritic plaque scores wherein arterial pathology outcomes were generally higher for those with neuritic plaques versus those with none, including for artery total area (p = 0.46), intima area (p = 0.21), intima-media area (p = 0.032), and %fibrosis (p = 0.003). Several arterial pathology measures differed by atherosclerosis severity such that higher values were observed with increasing severity of atherosclerosis for artery total area (p = 0.034), intima area (p = 0.01), intima-media area (p = 0.016), %fibrosis (p = 0.012), and %stenosis (p = 0.05). Arterial morphology measures did not differ for arteriolosclerosis severity, white matter rarefaction, nor cerebral amyloid angiopathy severity except for arterial media area being higher for those with mild CAA compared to those with none (p = 0.025).

## 4 Discussion and Conclusions

To our knowledge, this is the first study to investigate postmortem pathological correlates of antemortem 4D flow MRI outcomes in cerebral arteries. Our main findings suggest that 4D flow MRI outcomes in cerebral arteries reflect a combination of vascular morphology and pathological processes present in cerebral artery vascular remodeling that are consistent with established hemodynamic properties of cerebral arteries. The strongest associations between 4D flow MRI and vascular morphometry were observed when taken across different sized arterial segments (e.g., vascular area measures were highly correlated with 4D flow area, mean flow, and pulsatility index), except for fibrosis and stenosis which had much weaker antemortem-postmortem associations across cerebral artery segments. In contrast, associations across participants were weaker but were informative for understanding the extent to which 4D flow MRI reflects underlying vascular pathology, including some novel findings regarding 4D flow MRI area measures. Lastly, we also observed some associations and trends between cerebral artery morphology and 4D flow MRI with brain pathology outcomes that provide additional insights about how cerebral artery 4D flow MRI outcomes might reflect downstream brain pathology, and contextual information about hypothesized mechanisms regarding the impact of upstream vascular remodeling on downstream brain pathology. These results help clarify the interpretation of*in vivo*cerebral artery 4D flow MRI outcomes in the context of aging and cerebrovascular disease for larger clinical research studies.

A primary goal of this study was to better understand the extent to which cerebral artery 4D flow MRI outcomes were associated with local vascular pathology in the same segments of cerebral arteries. A challenge for interpreting*in vivo*4D flow MRI outcomes relates to challenges with imaging smaller vessels and measuring relatively small changes in vessels over time such as those that occur due to pathologic changes. For example, small vessels below the spatial resolution of the reconstructed image experience partial volume effects, but it is not clear whether in such scenarios measures such as lumen cross-sectional area and mean flow can be reliably estimated, if there is a lower limit for reliable estimation, or the extent to which 4D flow MRI is sensitive to small differences in luminal area between individuals and/or morphology changes associated with pathologic vascular remodeling processes such as fibrosis and stenosis. In our study, high positive correlations were observed across arterial segments between 4D flow area and mean flow, and several vascular pathology area measurements including cerebral arteries with total vascular areas in the 3–6 mm^2^range suggesting 4D flow MRI is sensitive to size differences between vessels including between small cerebral arteries. This finding is also consistent with 4D flow observations from a recent clinical study of 759 healthy older participants (Roberts, Peret, et al., 2023). These results are also consistent with expected hemodynamic relationships in cerebral arteries wherein larger arteries have higher blood flow than smaller vessels (Cebral et al., 2008). Associations between microscopic vascular area measurements and MRI area were lower for individual arteries and artery clusters across persons, which may suggest that 4D flow may be less sensitive to smaller changes observed between individuals or those associated with pathological processes. Interestingly, when looking at associations across participants with more granular artery clusters, 4D flow area indicated moderate to strong positive associations with %fibrosis, particularly for the internal carotid, vertebral, and middle-cerebral arteries. Similarly, we also observed moderate to strong positive associations between microscopic luminal area and %fibrosis, total vascular area, intima-media area, and intima area (i.e., plaque). We hypothesize that positive associations between 4D flow area, microscopically measured areas, and fibrosis may be due to stiffer (i.e., fibrotic) vessels having less*in vivo*contraction during diastole and less*ex vivo*shrinkage postmortem compared to healthy vessels, resulting in a more consistent and larger area when averaged over the cardiac cycle as is done when calculating 4D flow MRI cross-sectional area. This is also consistent with pathologic observations wherein fibrotic arteries had positive associations between luminal area and fibrosis, presumably due to fibrotic arteries undergoing less shrinkage following formalin fixation compared to arteries with preserved media and ample smooth muscle cells that would likely shrink and collapse more in the absence of blood pressure. Interestingly, we did not see much evidence of associations between vascular pathology measures and pulsatility index across participants, which was unexpected since pulsatility index is thought to be a measure of vascular stiffness/compliance. Taken together, these findings suggest that z-scored 4D flow area averaged across cerebral arteries might be a better measure of cerebral artery fibrosis than pulsatility index. Notably, 4D flow MRI studies of cerebral arteries often focus on mean blood flow and pulsatility index but could incorporate area measures which are also available but less frequently studied in the context of clinical aging and dementia research.

An additional challenge for contextualizing*in vivo*cerebral artery 4D flow MRI outcomes is that the process of vascular remodeling likely differs between large and small arteries, and we therefore might expect pathological correlates and the interpretation of 4D flow MRI outcomes to differ between large and small cerebral arteries. For example, coronary artery studies suggest these large arteries compensate for plaque deposition by increasing the circumference of the internal elastic lamina, which also corresponds to intimal-media thickening and thus the overall size of the artery increases up to a point in order to maintain luminal area and blood flow. One coronary artery study suggests the luminal area does not begin to decrease until ~40% stenosis, after which luminal area decreases with continued plaque deposition and stenosis (Glagov et al., 1987). In contrast, a 2014 study byGutierrez et al. (2014)suggests that cerebral arteries, particularly segments near the arterial circle (of Willis), experience a reduction in luminal area with nearly any level of stenosis, including for small cerebral arteries with luminal areas in the 1–2 mm^2^range with as little as 20% stenosis. In our study, we observed weak negative correlations between microscopically measured luminal area and stenosis for some arteries and observed stronger positive associations between stenosis and intima (i.e., plaque) area and intima-media thickness. Compared to stenosis, we observed generally stronger associations between pathology measures of media fibrosis and intima, lumen, and total vascular area, and generally weak associations between stenosis and fibrosis. This difference between our study and the previously published study may be due to the limited number of cases and the lower degree of stenosis observed in our study (most arterial segments in our study were <40% stenosed based on microscopic measurement). Concurrently, we observed differences in associations between 4D flow MRI and vascular pathology for clusters of large or small arteries. For example, associations between 4D flow and vascular morphometry measures were generally weaker for the PCA and ACA cluster compared to all other segments whereas associations were typically strongest for the larger vertebral and MCA cluster. Similar to vascular morphometry intercorrelations, associations between 4D flow MRI tended to be more strongly associated with fibrosis than stenosis. This difference in the strength of 4D flow MRI-pathology associations with differing artery sizes is likely due to a combination of differences in pathological processes between large and small cerebral arteries and challenges mentioned previously pertaining to imaging small vessels with 4D flow MRI. It is also possible that weaker associations between 4D flow MRI measures and stenosis might have been driven by differences in the sampling locations of arterial cross-sections between MR imaging and pathologic examination, despite using a common diagram when establishing these locations. These combined results suggest that clinical research studies utilizing 4D flow MRI should consider investigating means of 4D flow measures across similarly sized cerebral arteries as an optimal strategy to investigate associations between underlying cerebral artery pathology and aging and brain health outcomes.

Some studies suggest that pathologic changes in larger cerebral arteries contribute to downstream pathologic changes by allowing pulsatile flow and pulse pressure to reach downstream arterioles and capillary beds, which results in injury and neurovascular uncoupling that go on to affect cognitive function (de Montgolfier et al., 2019;Wahlin & Nyberg, 2019). While we did not see significant associations between cerebral artery 4D flow MRI outcomes and cerebrovascular pathology outcomes, there were trends worth noting. For example, mean cerebral artery blood flow trended lower for cases with mild-severe arteriolosclerosis and mild-moderate white matter rarefaction (only one case had severe white matter rarefaction). Additionally, there were trends such that higher cerebral artery 4D flow area and pulsatility index were observed for increasing severity of atherosclerosis assessed in the arterial circle (of Willis) during gross examination. We also observed significant associations between cerebral artery pathology and atherosclerosis wherein larger microscopically measured areas including total vascular area, intima area, and intima-media area, and higher degrees of stenosis and fibrosis were associated with greater severity of atherosclerosis. These findings are consistent with analyses suggesting that z-scored 4D flow area measures are indicative of a higher degree of arterial fibrosis. A related aside is that associations between cerebral artery pathology measures and qualitative pathologic assessment of atherosclerosis support the validity of our digital morphology measures. We did not observe any clear trends relating cerebral artery pathology to cerebral amyloid angiopathy, arteriolosclerosis, nor white matter rarefaction. We also did not observe any trends between 4D flow MRI outcomes and cerebral amyloid angiopathy. Taken together with the cerebral artery pathology-to-MRI correlates, these results might suggest that z-scored 4D flow area is indicative of local arterial fibrosis and atherosclerotic changes, whereas z-scored mean flow may be indicative of distal vascular brain pathology such as arteriolosclerosis and white matter rarefaction. These findings are also consistent with proposed mechanisms of leukoaraiosis that suggest pathologic vascular changes in the brain, particularly in white matter, are driven by reduced blood flow related to autoregulatory dysfunction (Pantoni & Garcia, 1997).

The common co-occurrence (Schneider et al., 2007) of and hypothesized causal or contributing links (DeCarli, 2003;de la Torre, 2010;Weller et al., 2009) between cerebrovascular disease and AD has led to increased interest in imaging vascular brain health during life. Particular interest in 4D flow MRI stems from the hypothesis that pulsatile blood flow drives clearance of neurodegenerative disease protein fragments, including the clearance of beta-amyloid fragments found in Alzheimer’s disease plaques (Mestre et al., 2020;Weller et al., 2009). In this study, we did not observe significant associations between antemortem 4D flow MRI and Alzheimer’s disease pathology including Thal beta-amyloid phase, Braak neurofibrillary tangle stage, nor neuritic plaque score. Similarly, we did not see meaningful differences in microscopically measured cerebral artery pathology across Thal phase or Braak stage but did see some significant differences in cerebral artery pathology with neuritic plaque scores such that those with higher neuritic plaque scores tended to have higher total vascular and intima-media areas and a higher degree of fibrosis compared to those with no neuritic plaque pathology. We also did not observe any trends or significant associations between cerebral artery pathology, 4D flow measures and cerebral amyloid angiopathy. These results should be interpreted cautiously given the relatively old age of the sample and the low number of control cases with low/no Alzheimer’s neuropathologic changes. Beyond AD pathology, we observed a significant association between lower mean blood flow and the presence of limbic or neocortical Lewy body pathology. We also observed significantly higher pulsatility index for individuals with medial temporal TDP-43 pathology compared to those with none. To date, few studies have investigated connections between Lewy body pathology and cerebrovascular pathology, especially cerebral artery pathology. Published studies have mixed findings with some reporting positive associations and others reporting negative or no associations (seeRaz et al., 2016for review). In addition, clinical studies using neuroimaging have also suggested patterns of both hypo- and hyperperfusion in those diagnosed with Lewy body dementia or Parkinson’s disease (Lin et al., 2017). Future clinicopathologic studies that include antemortem 4D flow MRI and neurodegenerative disease biomarkers with larger sample sizes will likely enable a better understanding of the interactions between cerebral artery pathology and hemodynamics, and cerebrovascular disease and brain pathology.

This study has several strengths and limitations. To our knowledge, this is the first study to investigate pathologic correlates of cerebral artery 4D flow MRI in cases with both antemortem imaging and postmortem pathologic characterization. Another key strength is the acquisition and pathologic characterization of the same vascular segments from multiple cerebral arteries for both MR imaging and pathology. In addition, the use of quantitative measures derived from digital pathology images enabled us to study key aspects such as fibrosis in a more granular way than qualitative assessments allow. A key limitation of the study is the relatively small (n = 20) convenience sample primarily comprising individuals with clinical impairment and some degree of Alzheimer’s disease pathology. However, it is also notable that this sample did have variability in aspects of cerebrovascular disease. We also did not correct for multiple comparisons in this exploratory study, and it is possible that the associations reported may not remain significant in future studies. In addition, the nature of antemortem-postmortem studies creates time gaps between antemortem imaging and pathologic assessment that add additional variability to these comparisons. Regardless of these limitations, we believe this study provides novel insights into the pathological correlates of cerebral artery 4D flow MRI.

In summary, our preliminary findings from 20 initial cases suggest 4D flow MRI provides useful information about cerebral artery hemodynamics that correlate with underlying vascular pathology such as fibrosis and atherosclerosis that contribute to cerebrovascular disease. Future clinicopathologic studies with a larger number of cases, including a larger number of control cases, are needed to better understand the nuanced interpretations of 4D flow MRI outcomes in clinical aging and dementia research studies.

## Supplementary Material

Supplementary Material

## Data Availability

MRI and pathology data are available for qualified investigators upon approved request to the Wisconsin Alzheimer’s Disease Research Center (https://wrap.wisc.edu/data-requests/). A validated 4D flow MRI data processing tool used in this study can be found online (https://github.com/uwmri/QVT).
