# Supplementary material for: Cerebral artery and brain pathology correlates of antemortem cerebral artery 4D flow MRI
Source: Imaging Neurosci (Camb). 2024 Oct 25;2:imag-2-00322. doi: 10.1162/imag_a_00322 (PMC11908694; doi:10.1162/imag_a_00322)
Supplement: Supplementary Material [file imag_a_00322-supp.pdf]

Supplemental Tables and Figures

Supplemental Table 1: Artery-level means and standard deviations for all cerebral artery segments

Pathology/MRI	Variable	ICA-L	Basilar	Vertebral-L	Vertebral-R	MCA-M1-L	MCA-M1-R	PCA-P2-L	PCA-P2-R	PCA-P1-L	PCA-P1-R
	n observations	18	17	14	18	20	17	18	17	18	18
Pathology	Total Vascular Area	8.10 (3.91)	6.14 (2.86)	5.10 (3.79)	4.22 (2.71)	4.09 (1.65)	2.87 (1.28)	3.80 (2.55)	2.78 (1.48)	2.93 (1.73)	1.99 (0.750)
Pathology	Lumen Area	4.25 (2.77)	3.74 (2.29)	2.99 (2.42)	2.53 (1.78)	2.48 (1.31)	1.54 (0.870)	2.21 (1.73)	1.45 (0.915)	1.47 (1.19)	1.12 (0.641)
Pathology	Intima Area	1.90 (1.82)	0.767 (0.717)	0.991 (1.38)	0.727 (0.980)	0.525 (0.544)	0.521 (0.450)	0.693 (0.592)	0.462 (0.592)	0.557 (0.395)	0.211 (0.0906)
Pathology	Media Area	1.85 (0.632)	1.56 (0.569)	1.05 (0.447)	0.909 (0.354)	1.04 (0.390)	0.780 (0.458)	0.851 (0.501)	0.837 (0.340)	0.868 (0.469)	0.628 (0.182)
Pathology	Intima-Media Area	3.75 (1.93)	2.32 (0.824)	2.05 (1.61)	1.64 (1.15)	1.56 (0.615)	1.30 (0.585)	1.54 (0.899)	1.30 (0.793)	1.42 (0.745)	0.838 (0.218)
Pathology	Intima-Media Thickness	0.454 (0.203)	0.322 (0.0969)	0.294 (0.122)	0.259 (0.113)	0.261 (0.0846)	0.260 (0.0897)	0.261 (0.0913)	0.264 (0.116)	0.290 (0.106)	0.207 (0.0616)
Pathology	Media Smooth Muscle Area	0.841 (0.334)	0.825 (0.335)	0.513 (0.284)	0.472 (0.312)	0.535 (0.207)	0.315 (0.160)	0.504 (0.467)	0.415 (0.146)	0.464 (0.252)	0.389 (0.111)
Pathology	%Fibrosis	49.6 (17.0)	40.6 (19.3)	42.9 (26.6)	43.1 (26.0)	41.0 (15.2)	50.6 (21.9)	39.3 (29.1)	44.0 (17.4)	32.4 (19.2)	33.3 (12.9)
Pathology	%Stenosis	27.9 (20.7)	18.8 (13.7)	22.1 (11.9)	21.5 (14.7)	18.1 (13.0)	25.2 (18.5)	23.7 (13.8)	23.7 (20.4)	32.0 (18.8)	18.8 (12.7)
MRI	MRI Area	17.1 (4.30)	8.38 (2.06)	6.79 (1.78)	8.02 (3.42)	7.00 (1.26)	4.85 (0.905)	4.86 (0.852)	5.40 (1.58)	5.04 (0.718)	4.49 (1.69)
MRI	MRI Mean Flow	3.46 (1.09)	1.74 (0.685)	1.11 (0.435)	1.32 (0.566)	1.89 (0.689)	0.755 (0.293)	0.783 (0.280)	0.800 (0.296)	0.793 (0.274)	1.14 (0.518)
MRI	MRI Pulsatility Index	1.25 (0.315)	1.33 (0.268)	1.37 (0.493)	1.54 (0.941)	1.38 (0.424)	1.61 (0.482)	1.64 (0.452)	1.53 (0.527)	1.78 (0.670)	1.62 (0.448)

Values are reported as mean (SD)

All areas are reported in mm^2; intima-media thickness is reported in mm; MRI mean flow is reported in mL/cycle

Supplemental Table 2: Associations between vessel pathology and 4D Flow MRI and neuropathological examination

Vascular Measure	Brain Pathology Assessment	p	R^2	R_{adj}^2
za(Total Vascular Area)	Thal Phase	0.69	0.13	-0.1
za(Lumen Area)	Thal Phase	0.78	0.1	-0.14
za(Intima Area)	Thal Phase	0.18	0.32	0.14
za(Media Area)	Thal Phase	0.18	0.32	0.14
za(Intima-Media Area)	Thal Phase	0.24	0.29	0.1
**za(Intima-Media Thickness)**	**Thal Phase**	**0.05**	**0.45**	**0.3**
za(Media Smooth Muscle Area)	Thal Phase	0.25	0.28	0.1
%Fibrosis	Thal Phase	0.17	0.33	0.15
%Stenosis	Thal Phase	0.13	0.36	0.19
za(MRI Area)	Thal Phase	0.63	0.15	-0.08
za(MRI Mean Flow)	Thal Phase	0.66	0.14	-0.09
MRI Pulsatility Index	Thal Phase	0.83	0.09	-0.15
za(Total Vascular Area)	Braak NFT Stage	0.89	0.11	-0.21
za(Lumen Area)	Braak NFT Stage	0.84	0.12	-0.19
za(Intima Area)	Braak NFT Stage	0.54	0.23	-0.04
za(Media Area)	Braak NFT Stage	0.8	0.14	-0.17
za(Intima-Media Area)	Braak NFT Stage	0.52	0.24	-0.03
za(Intima-Media Thickness)	Braak NFT Stage	0.25	0.35	0.12
za(Media Smooth Muscle Area)	Braak NFT Stage	0.6	0.21	-0.07
%Fibrosis	Braak NFT Stage	0.44	0.27	0.01
%Stenosis	Braak NFT Stage	0.1	0.45	0.26
za(MRI Area)	Braak NFT Stage	0.5	0.25	-0.02
za(MRI Mean Flow)	Braak NFT Stage	0.24	0.36	0.13
MRI Pulsatility Index	Braak NFT Stage	0.28	0.33	0.1
**za(Total Vascular Area)**	**Neuritic Plaque Score**	**0.046**	**0.38**	**0.27**
za(Lumen Area)	Neuritic Plaque Score	0.21	0.24	0.1
**za(Intima Area)**	**Neuritic Plaque Score**	**0.021**	**0.45**	**0.34**
za(Media Area)	Neuritic Plaque Score	0.06	0.36	0.25
**za(Intima-Media Area)**	**Neuritic Plaque Score**	**0.032**	**0.41**	**0.3**
za(Intima-Media Thickness)	Neuritic Plaque Score	0.35	0.18	0.03
za(Media Smooth Muscle Area)	Neuritic Plaque Score	0.26	0.22	0.07
**%Fibrosis**	**Neuritic Plaque Score**	**0.003**	**0.57**	**0.48**
%Stenosis	Neuritic Plaque Score	0.78	0.06	-0.11
za(MRI Area)	Neuritic Plaque Score	0.34	0.18	0.03
za(MRI Mean Flow)	Neuritic Plaque Score	0.74	0.07	-0.1
MRI Pulsatility Index	Neuritic Plaque Score	0.44	0.15	-0.01
za(Total Vascular Area)	Lewy Bodies	0.77	0.03	-0.08
za(Lumen Area)	Lewy Bodies	0.85	0.02	-0.1
za(Intima Area)	Lewy Bodies	0.29	0.14	0.03
za(Media Area)	Lewy Bodies	0.27	0.14	0.04
za(Intima-Media Area)	Lewy Bodies	0.78	0.03	-0.09
za(Intima-Media Thickness)	Lewy Bodies	0.88	0.01	-0.1
za(Media Smooth Muscle Area)	Lewy Bodies	0.76	0.03	-0.08
%Fibrosis	Lewy Bodies	0.76	0.03	-0.08
%Stenosis	Lewy Bodies	0.45	0.09	-0.02
za(MRI Area)	Lewy Bodies	0.16	0.2	0.1

**za(MRI Mean Flow)**	**Lewy Bodies**	**0.006**	**0.45**	**0.39**
MRI Pulsatility Index	Lewy Bodies	0.27	0.14	0.04
za(Total Vascular Area)	TDP-43 (Amygdala)	0.76	0.01	-0.05
za(Lumen Area)	TDP-43 (Amygdala)	0.45	0.03	-0.02
za(Intima Area)	TDP-43 (Amygdala)	0.82	0	-0.05
za(Media Area)	TDP-43 (Amygdala)	0.71	0.01	-0.05
za(Intima-Media Area)	TDP-43 (Amygdala)	0.69	0.01	-0.05
za(Intima-Media Thickness)	TDP-43 (Amygdala)	0.45	0.03	-0.02
za(Media Smooth Muscle Area)	TDP-43 (Amygdala)	0.21	0.09	0.03
%Fibrosis	TDP-43 (Amygdala)	0.48	0.03	-0.03
%Stenosis	TDP-43 (Amygdala)	0.25	0.07	0.02
za(MRI Area)	TDP-43 (Amygdala)	0.41	0.04	-0.01
za(MRI Mean Flow)	TDP-43 (Amygdala)	0.15	0.11	0.06
MRI Pulsatility Index	TDP-43 (Amygdala)	0.27	0.07	0.01
za(Total Vascular Area)	TDP-43 (MTL)	0.93	0	-0.06
za(Lumen Area)	TDP-43 (MTL)	0.63	0.01	-0.04
za(Intima Area)	TDP-43 (MTL)	0.94	0	-0.06
za(Media Area)	TDP-43 (MTL)	0.37	0.05	-0.01
za(Intima-Media Area)	TDP-43 (MTL)	0.64	0.01	-0.04
za(Intima-Media Thickness)	TDP-43 (MTL)	0.54	0.02	-0.03
za(Media Smooth Muscle Area)	TDP-43 (MTL)	0.11	0.14	0.09
%Fibrosis	TDP-43 (MTL)	0.8	0	-0.05
%Stenosis	TDP-43 (MTL)	0.6	0.02	-0.04
za(MRI Area)	TDP-43 (MTL)	0.19	0.1	0.04
za(MRI Mean Flow)	TDP-43 (MTL)	0.2	0.09	0.04
**MRI Pulsatility Index**	**TDP-43 (Hippocampus)**	**0.017**	**0.28**	**0.24**
za(Total Vascular Area)	Cerebral Amyloid Angiopathy	0.68	0.09	-0.09
za(Lumen Area)	Cerebral Amyloid Angiopathy	0.79	0.06	-0.11
za(Intima Area)	Cerebral Amyloid Angiopathy	0.4	0.17	0.01
**za(Media Area)**	**Cerebral Amyloid Angiopathy**	**0.025**	**0.43**	**0.33**
za(Intima-Media Area)	Cerebral Amyloid Angiopathy	0.26	0.22	0.07
za(Intima-Media Thickness)	Cerebral Amyloid Angiopathy	0.19	0.25	0.11
za(Media Smooth Muscle Area)	Cerebral Amyloid Angiopathy	0.52	0.13	-0.03
%Fibrosis	Cerebral Amyloid Angiopathy	0.66	0.09	-0.08
%Stenosis	Cerebral Amyloid Angiopathy	0.43	0.15	0
za(MRI Area)	Cerebral Amyloid Angiopathy	0.49	0.14	-0.03
za(MRI Mean Flow)	Cerebral Amyloid Angiopathy	0.56	0.12	-0.05
MRI Pulsatility Index	Cerebral Amyloid Angiopathy	0.99	0.01	-0.18
**za(Total Vascular Area)**	**Atherosclerosis**	**0.034**	**0.41**	**0.3**
za(Lumen Area)	Atherosclerosis	0.2	0.24	0.1
**za(Intima Area)**	**Atherosclerosis**	**0.01**	**0.5**	**0.41**
za(Media Area)	Atherosclerosis	0.15	0.27	0.14
**za(Intima-Media Area)**	**Atherosclerosis**	**0.016**	**0.47**	**0.36**
za(Intima-Media Thickness)	Atherosclerosis	0.1	0.32	0.19
za(Media Smooth Muscle Area)	Atherosclerosis	0.2	0.25	0.11
**%Fibrosis**	**Atherosclerosis**	**0.012**	**0.49**	**0.39**
**%Stenosis**	**Atherosclerosis**	**0.05**	**0.38**	**0.26**
za(MRI Area)	Atherosclerosis	0.44	0.15	-0.01
za(MRI Mean Flow)	Atherosclerosis	0.57	0.12	-0.05
MRI Pulsatility Index	Atherosclerosis	0.66	0.09	-0.08

za(Total Vascular Area)	Arteriolosclerosis	0.64	0.1	-0.07
za(Lumen Area)	Arteriolosclerosis	0.77	0.07	-0.11
za(Intima Area)	Arteriolosclerosis	0.39	0.17	0.01
za(Media Area)	Arteriolosclerosis	0.93	0.03	-0.16
za(Intima-Media Area)	Arteriolosclerosis	0.45	0.15	-0.01
za(Intima-Media Thickness)	Arteriolosclerosis	0.4	0.16	0.01
za(Media Smooth Muscle Area)	Arteriolosclerosis	0.65	0.1	-0.07
%Fibrosis	Arteriolosclerosis	0.83	0.05	-0.13
%Stenosis	Arteriolosclerosis	0.4	0.16	0.01
za(MRI Area)	Arteriolosclerosis	0.5	0.13	-0.03
za(MRI Mean Flow)	Arteriolosclerosis	0.43	0.15	-0.01
MRI Pulsatility Index	Arteriolosclerosis	0.67	0.09	-0.08
za(Total Vascular Area)	White Matter Rarefaction	0.6	0.11	-0.06
za(Lumen Area)	White Matter Rarefaction	0.61	0.1	-0.06
za(Intima Area)	White Matter Rarefaction	0.55	0.12	-0.04
za(Media Area)	White Matter Rarefaction	0.23	0.23	0.09
za(Intima-Media Area)	White Matter Rarefaction	0.56	0.12	-0.05
za(Intima-Media Thickness)	White Matter Rarefaction	0.54	0.12	-0.04
za(Media Smooth Muscle Area)	White Matter Rarefaction	0.31	0.2	0.04
%Fibrosis	White Matter Rarefaction	0.78	0.06	-0.11
%Stenosis	White Matter Rarefaction	0.36	0.18	0.02
za(MRI Area)	White Matter Rarefaction	0.82	0.06	-0.12
za(MRI Mean Flow)	White Matter Rarefaction	0.16	0.27	0.13
MRI Pulsatility Index	White Matter Rarefaction	0.68	0.09	-0.08

Supplemental Figure 1: CONSORT diagram for included cases and cerebral artery segments

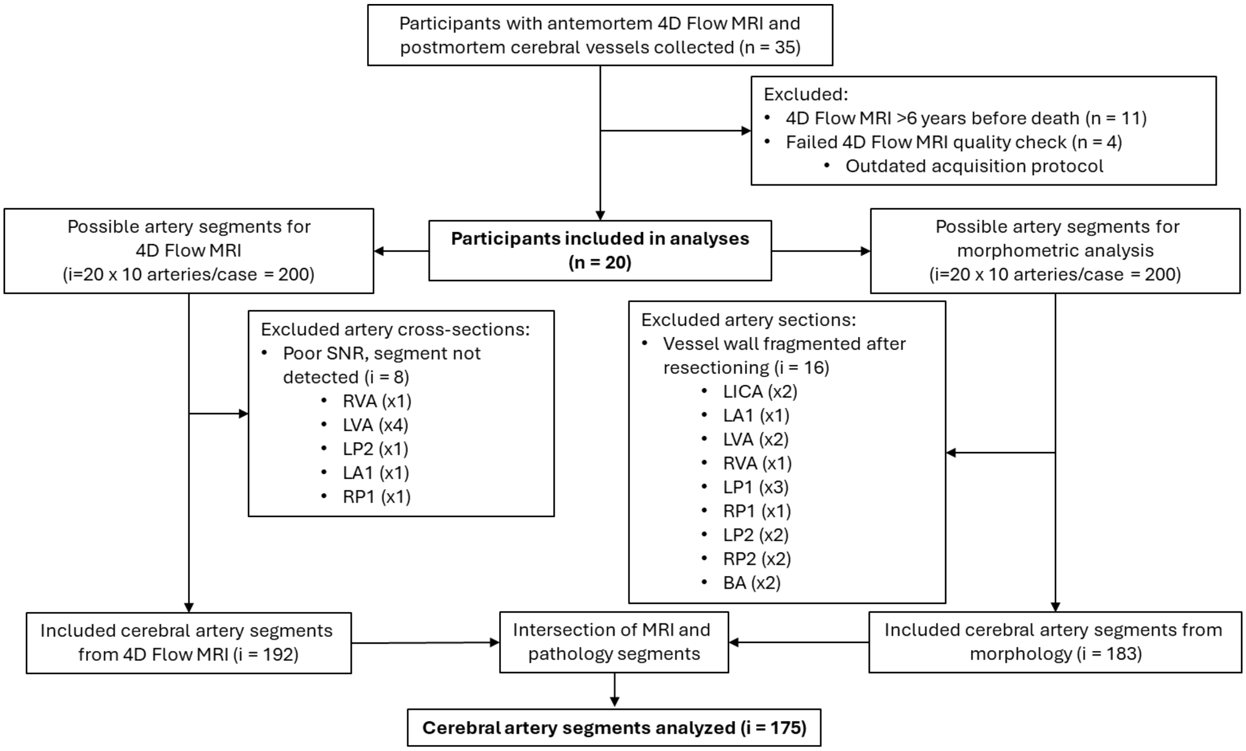

LICA = left internal carotid artery; BA = basilar artery; LP1 = left posterior communicating artery, pre-communicating segment; LP2 = left posterior communicating artery, post-communicating segment; RP1 = right posterior communicating artery, pre-communicating segment; RP2 = right posterior communicating artery, post-communicating segment; LA1 = left anterior cerebral artery; LM1 = left middle cerebral artery; RVA = right vertebral artery; LVA = left vertebral artery

Supplemental Figure 2: 4D Flow MRI quantification example

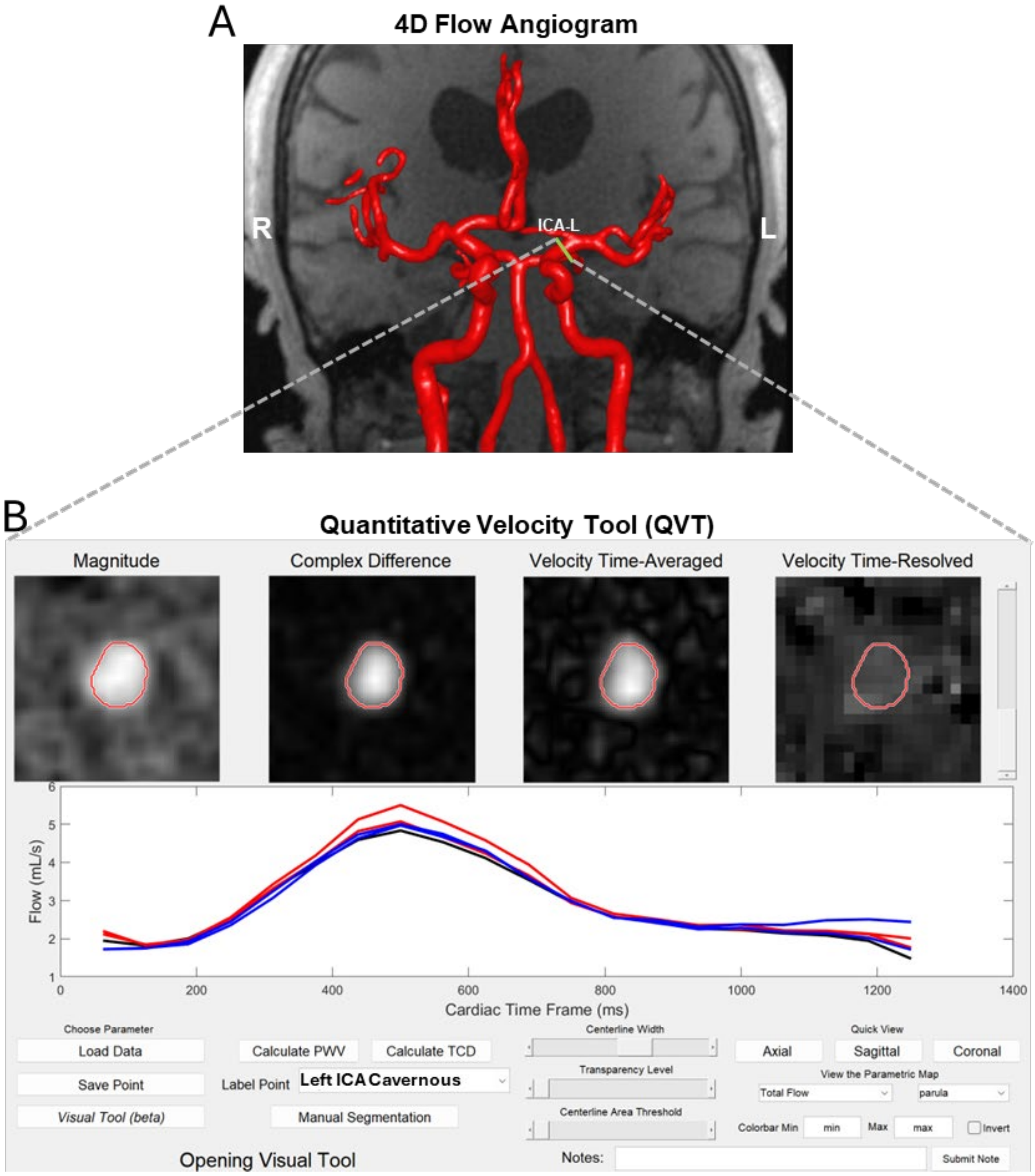

An annotated angiogram (panel A) and cerebral blood flow dynamics (panel B) in the left cavernous internal carotid artery (ICA) from an exemplar PC VIPR 4D flow MRI scan assessed by the validated quantitative velocity tool (QVT; https://github.com/uwmri/QVT). The tool enables visualization of different aspects of each cerebral artery cross-section, blood flow over time, and calculates variables of interest (pulsatility index, mean flow, area) for each artery cross-section. The tool is also used for visual quality control of the 4D flow MR images and flow data.

Supplemental Figure 3: Vascular annotation and segmentation for cerebral artery morphology measures

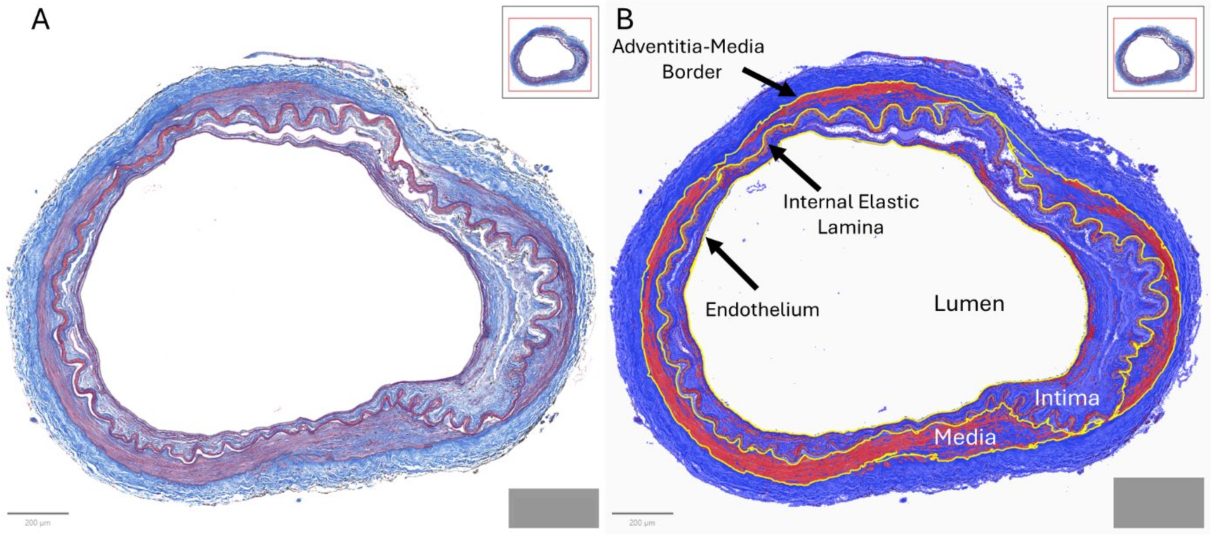

Panel A is an example of a cerebral artery (left anterior cerebral artery, A1 segment) used for morphometry. It reveals atherosclerotic changes affecting the intima and the media. Original digital scan at 20x magnification. Panel B reveals annotations with yellow lines, outlining from internal to external, the vascular lumen, the internal elastic lamina, and the external border of the media. Thus, areas within and between the lines of annotation were used to measure luminal area, areas of the intima, and media respectively. For all intents and purposes, the normal intima has a negligible area while this area expands with the development of the intimal plaque of atherosclerosis. For changes in the media, percent fibrosis was defined as the percentage area of collagen/fibrous tissue, stained blue with trichrome, divided by the total area of the media. Percent stenosis was defined as the area of expanded intima divided by the area encompassed by the internal elastic lamina. Panel B also shows the pixel classification of smooth muscle cells (red) and fibrous/collagenous tissue (blue).

Supplemental Figure 4: Z-scoring and averaging approach for vascular morphometry and 4D flow outcomes

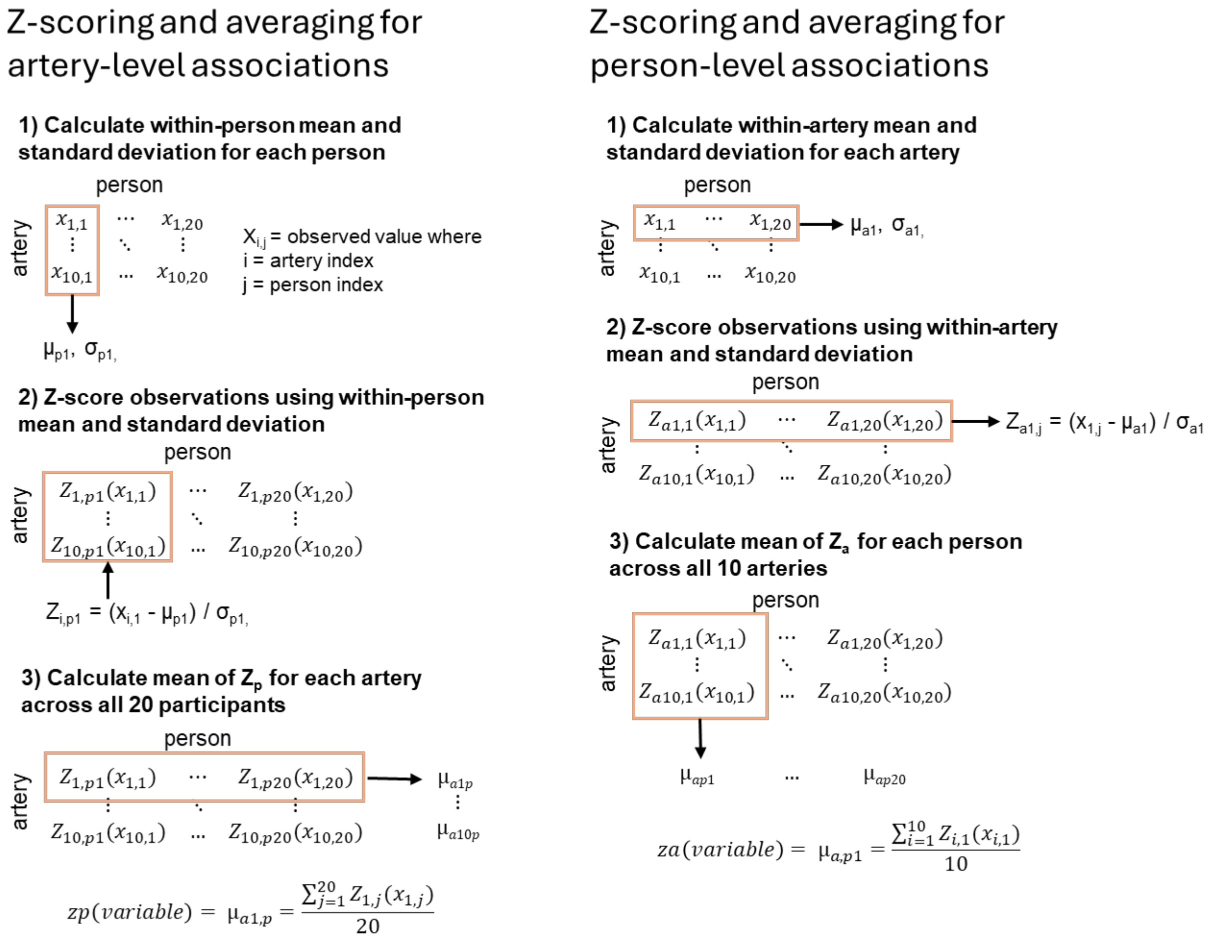

Diagrams show how the zp(*variable*) and za(*variable*) metrics were generated for analyses in sections 3.3 and 3.4, where *variable* represents vascular morphometry or 4D flow MRI measures. The left side of the figure indicates the process that was used to generate values used to investigate correlations between vascular morphometry and 4D flow measures across arteries (i.e., artery-level correlations). The right side of the figure indicates the process that was used to generate values to investigate correlations between vascular morphometry and 4D flow measures across participants (i.e., person-level correlations).

Supplemental Figure 5: Example of paired differences cluster visualization

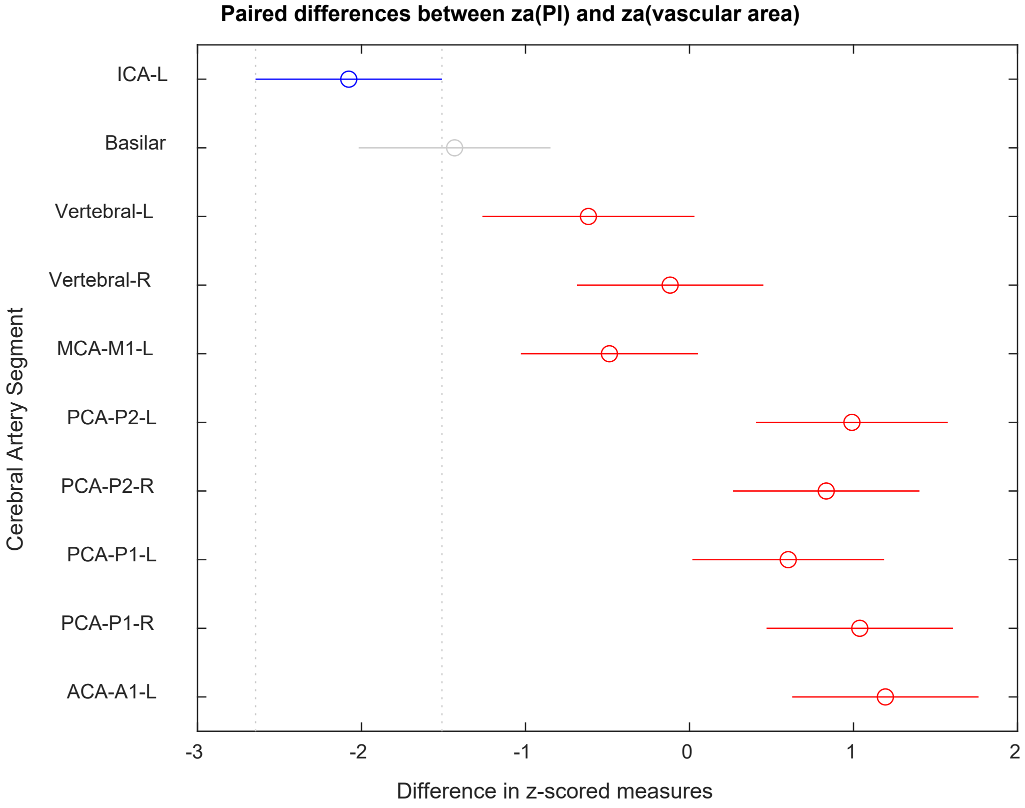

An example of how ANOVAs of paired differences were used to establish cerebral artery clusters for analysis. In this example, pulsatility index from MRI and vascular area from morphology were first z-scored using artery-level means and standard deviations, and then subtracted to establish differences in the z-scored measures. An ANOVA was then performed to determine if the difference in z-scored measures was significantly different between cerebral artery segments. In this example, paired differences between za(pulsatility index) and za(vascular area) are significantly different between arteries of differing sizes but are similar for similarly sized arteries. These tests were repeated for all pairs of 4D flow and morphology measures.

Supplemental Figure 6: Correlograms for each individual cerebral artery segment

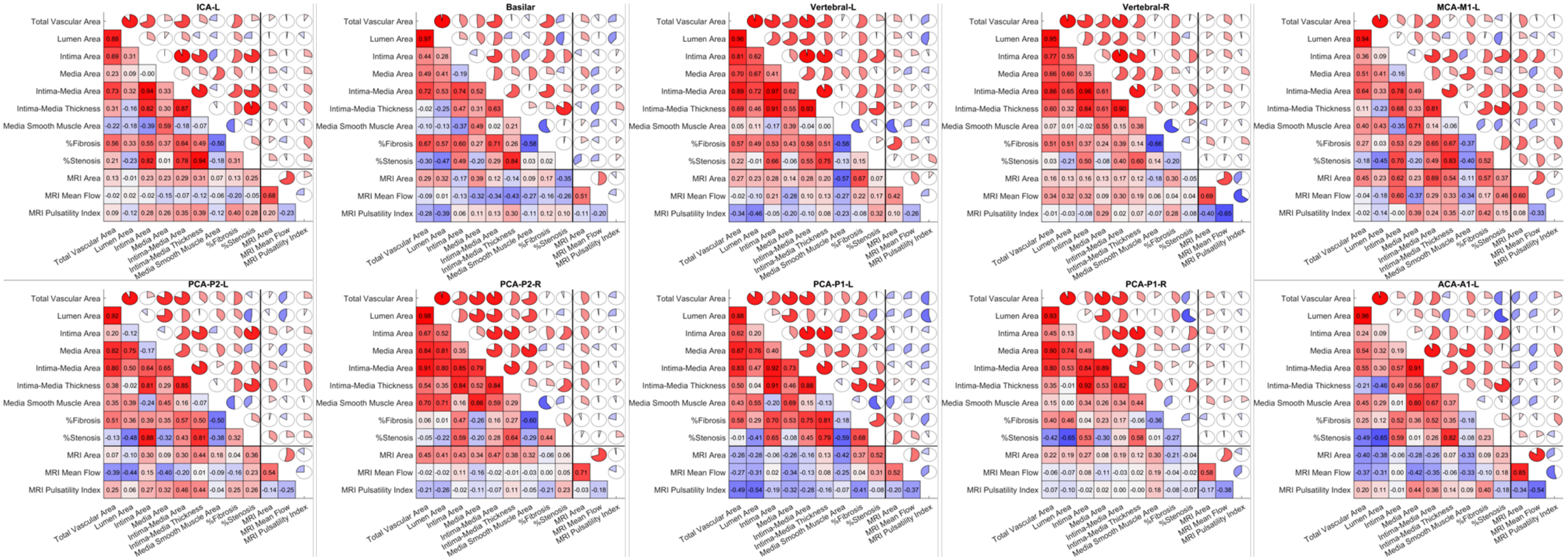

Correlograms for all individual artery segments ordered left-to-right and top-to-bottom by artery size based on total vascular area from vascular morphology.

Supplemental Figure 7: Scatter plots for artery-level comparisons

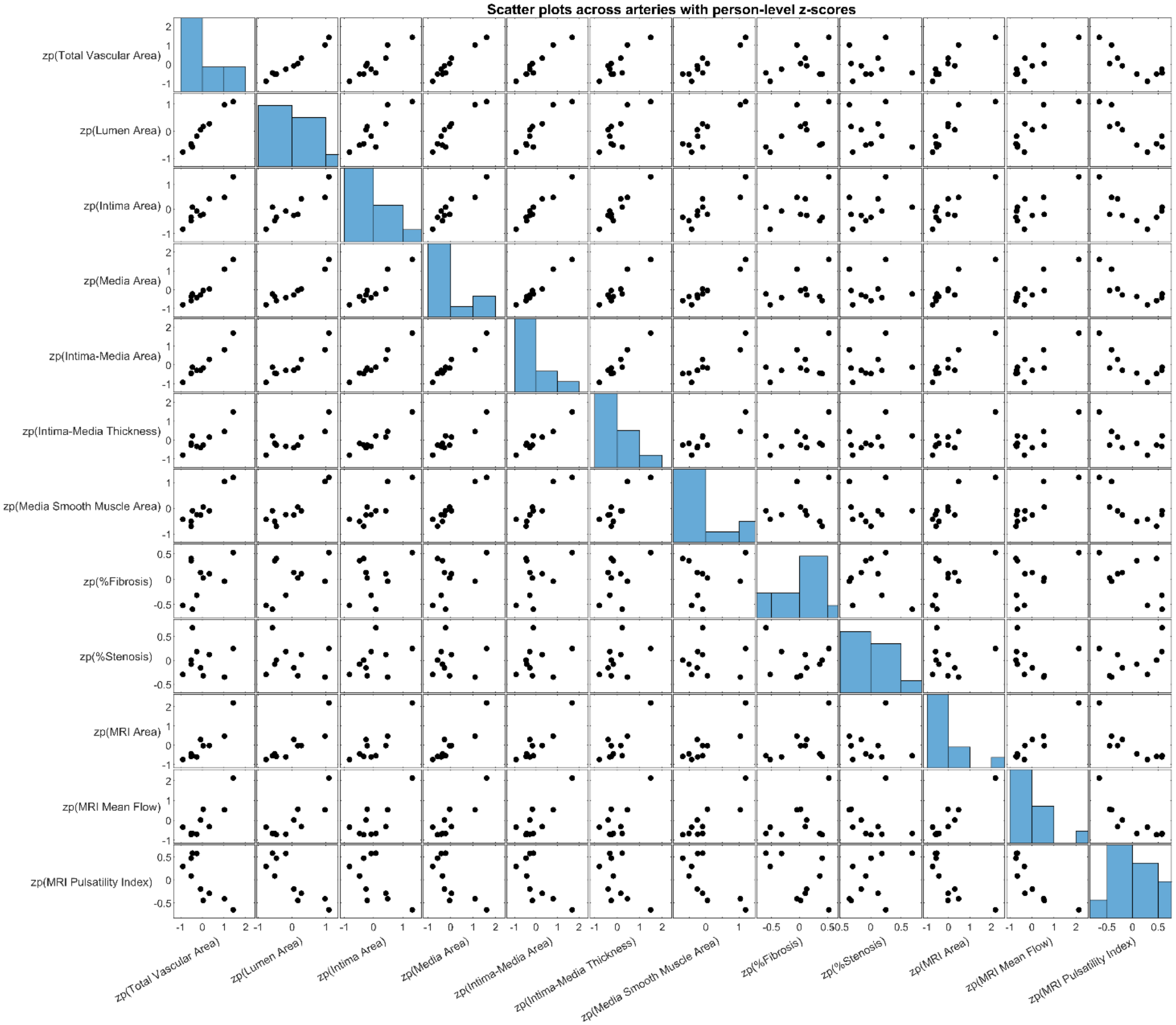

zp indicates measures were z-scored at the person level.

Supplemental Figure 8: Scatter plots for person-level comparisons

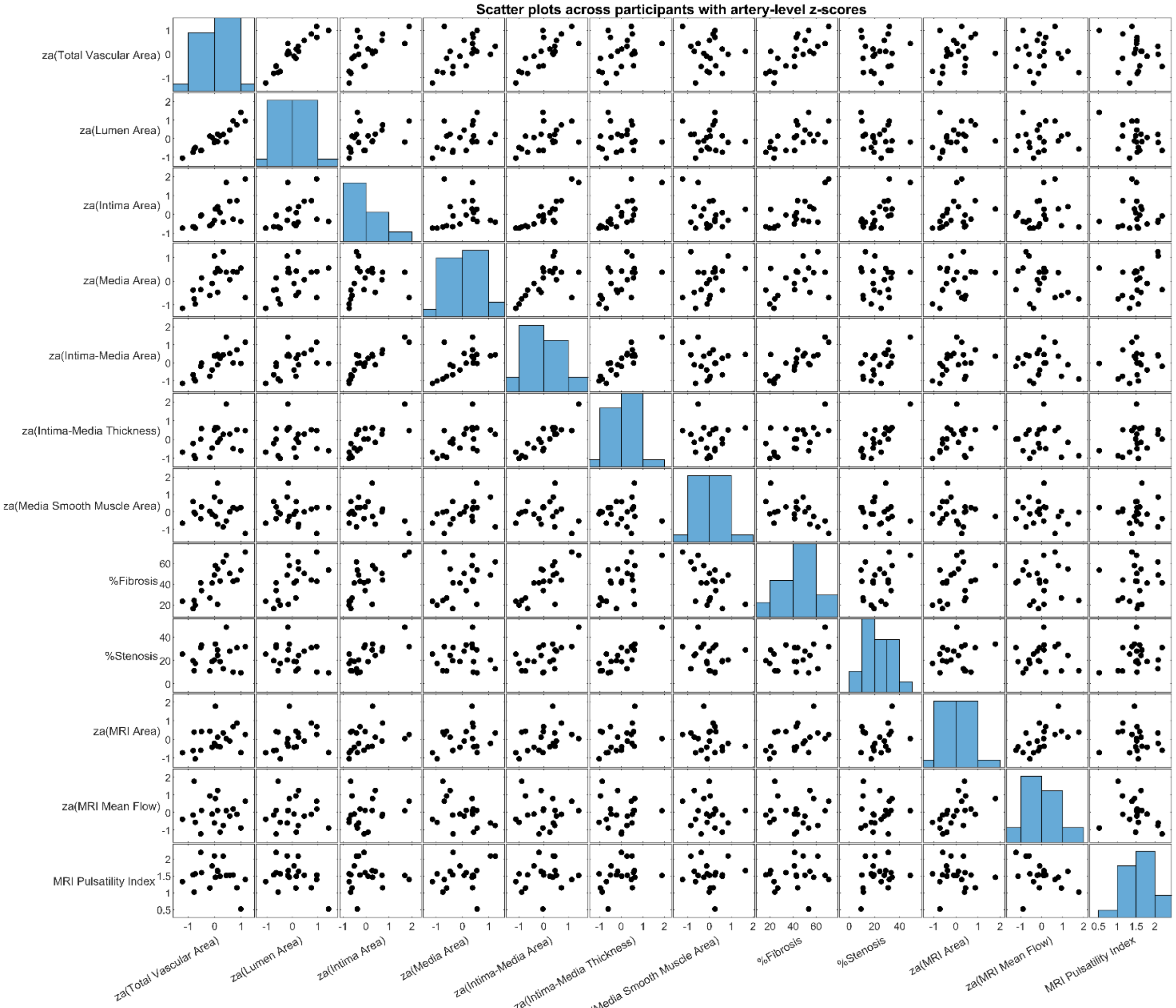

za indicates measures were z-scored at the artery level.

Supplemental Figure 9: Scatter plots by cerebral artery clusters

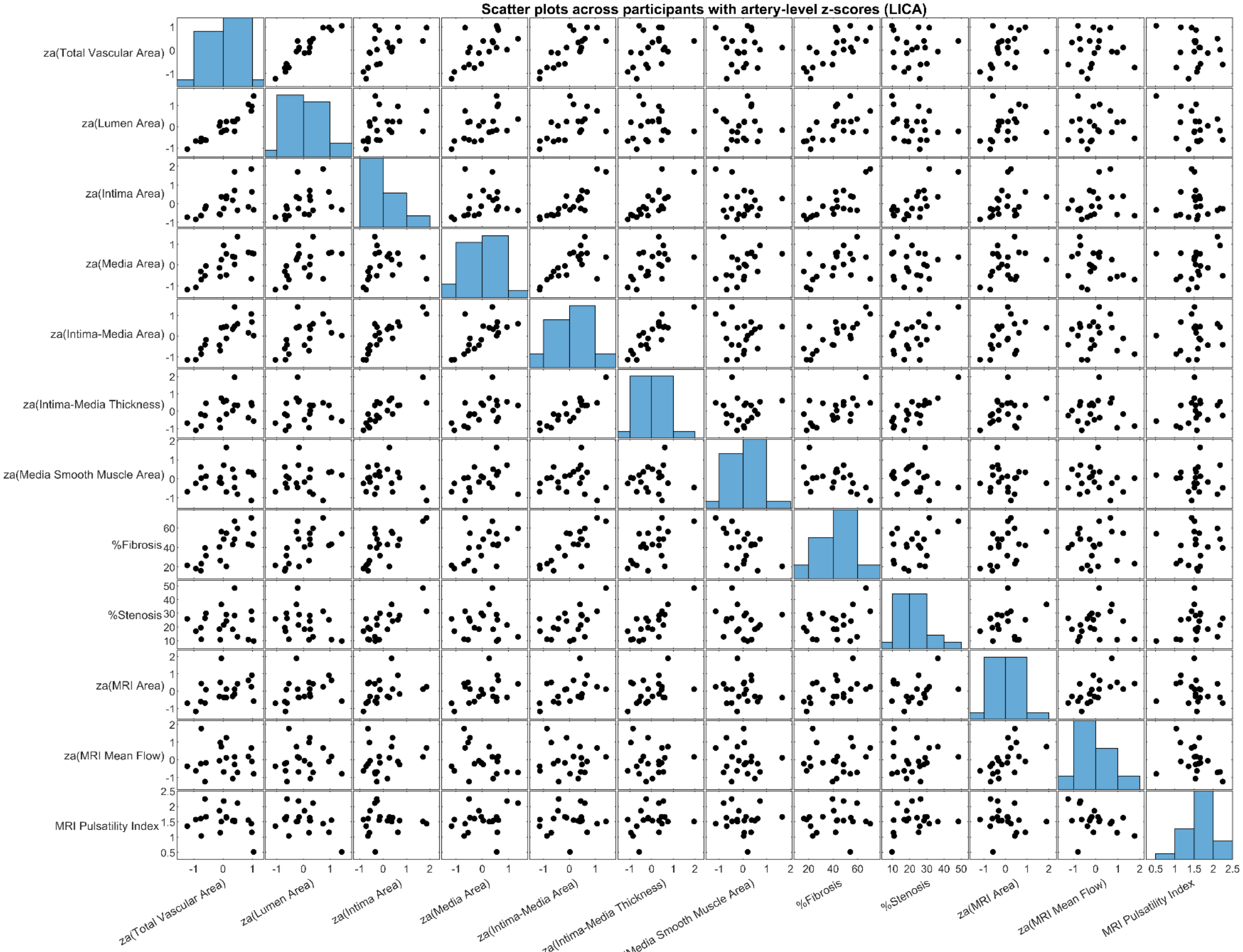

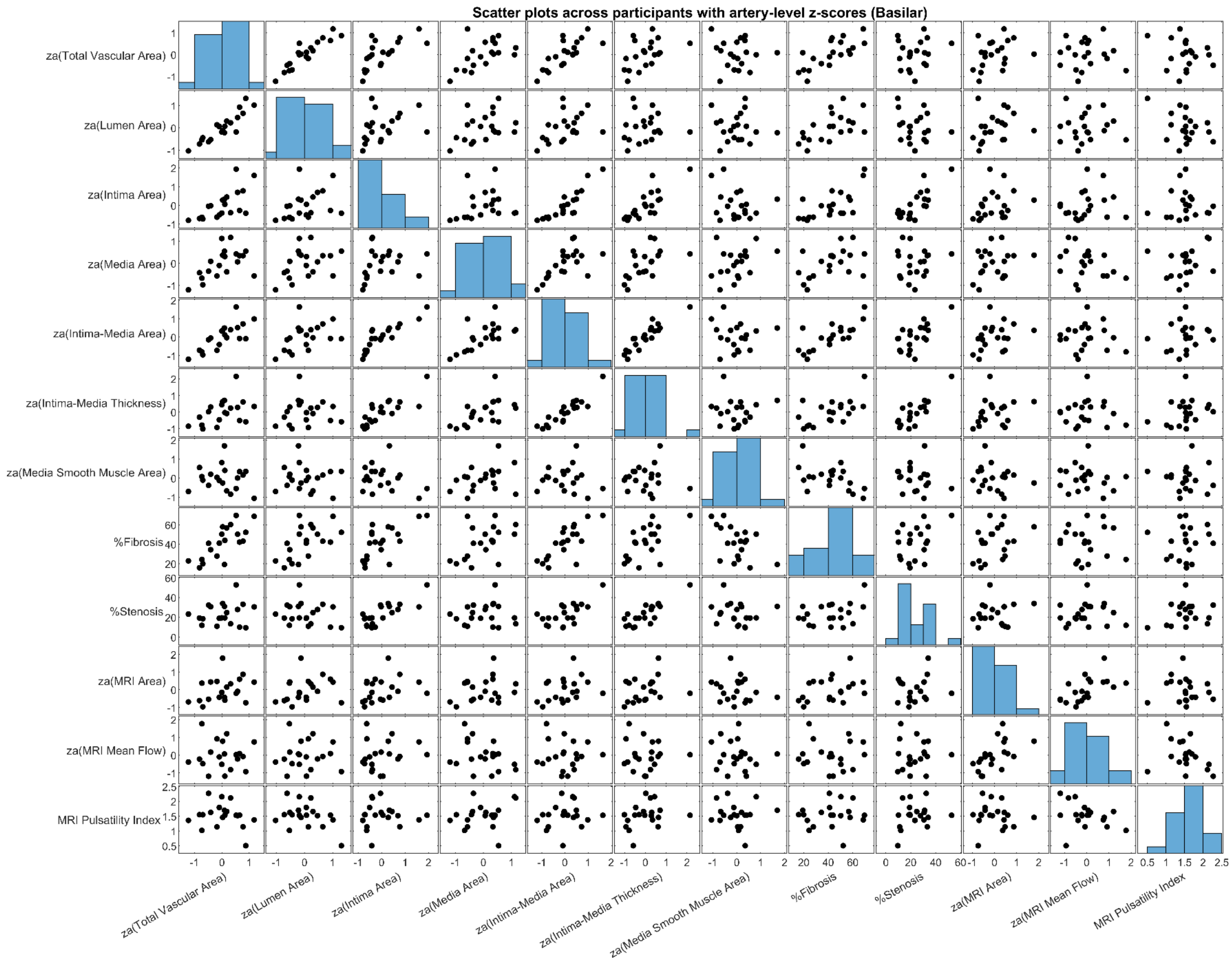

Scatter plots across participants with artery-level z-scores (Basilar)
za(Total Vascular Area)
za(Lumen Area)
za(Intima Area)
za(Media Area)
za(Intima-Media Area)
za(Intima-Media Thickness)
za(Media Smooth Muscle Area)
%Fibrosis
%Stenosis
za(MRI Area)
za(MRI Mean Flow)
MRI Pulsatility Index

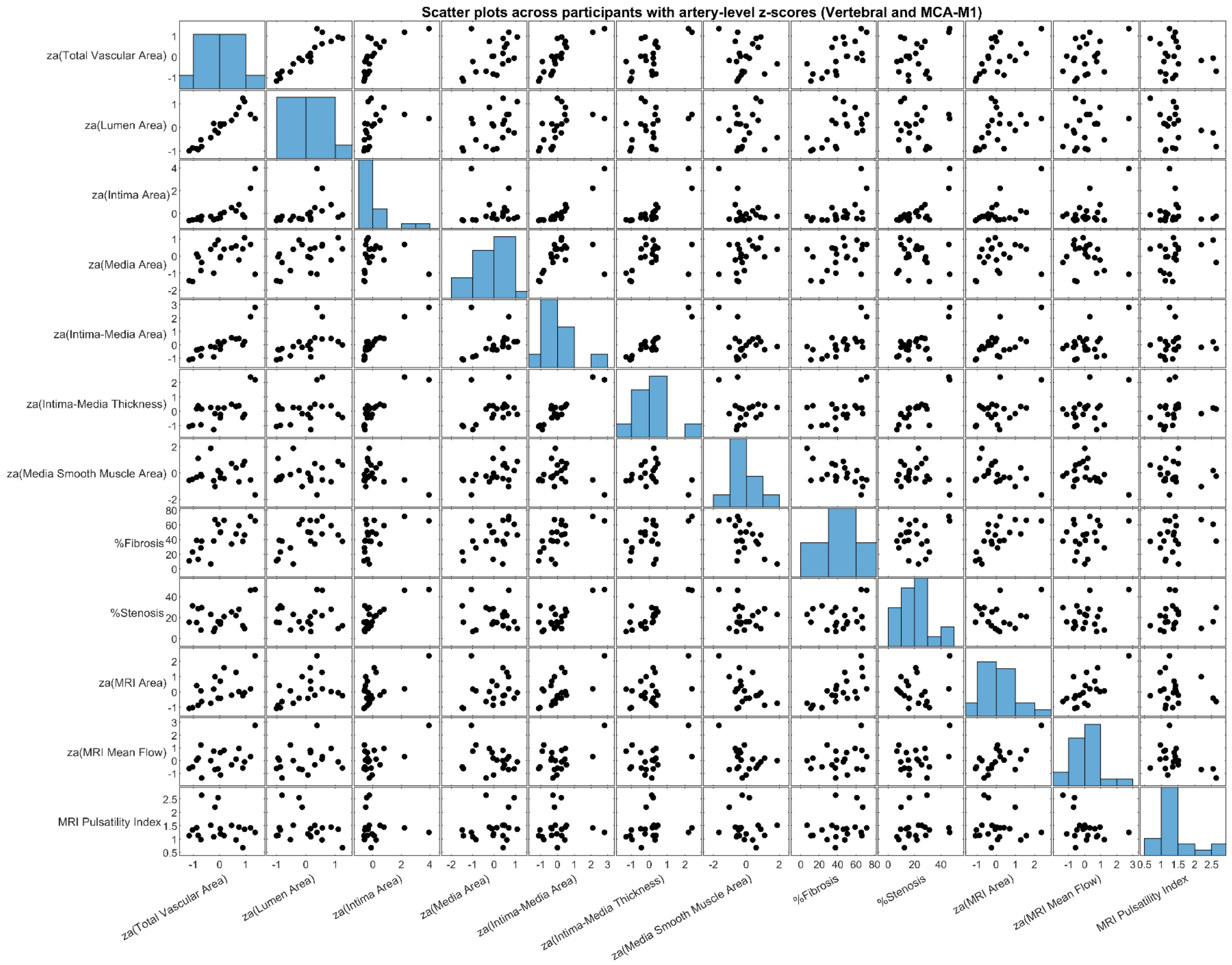

Scatter plots across participants with artery-level z-scores (Vertebral and MCA-M1)
za(Total Vascular Area)
za(Lumen Area)
za(Intima Area)
za(Media Area)
za(Intima-Media Area)
za(Intima-Media Thickness)
za(Media Smooth Muscle Area)
%Fibrosis
%Stenosis
za(MRI Area)
za(MRI Mean Flow)
MRI Pulsatility Index

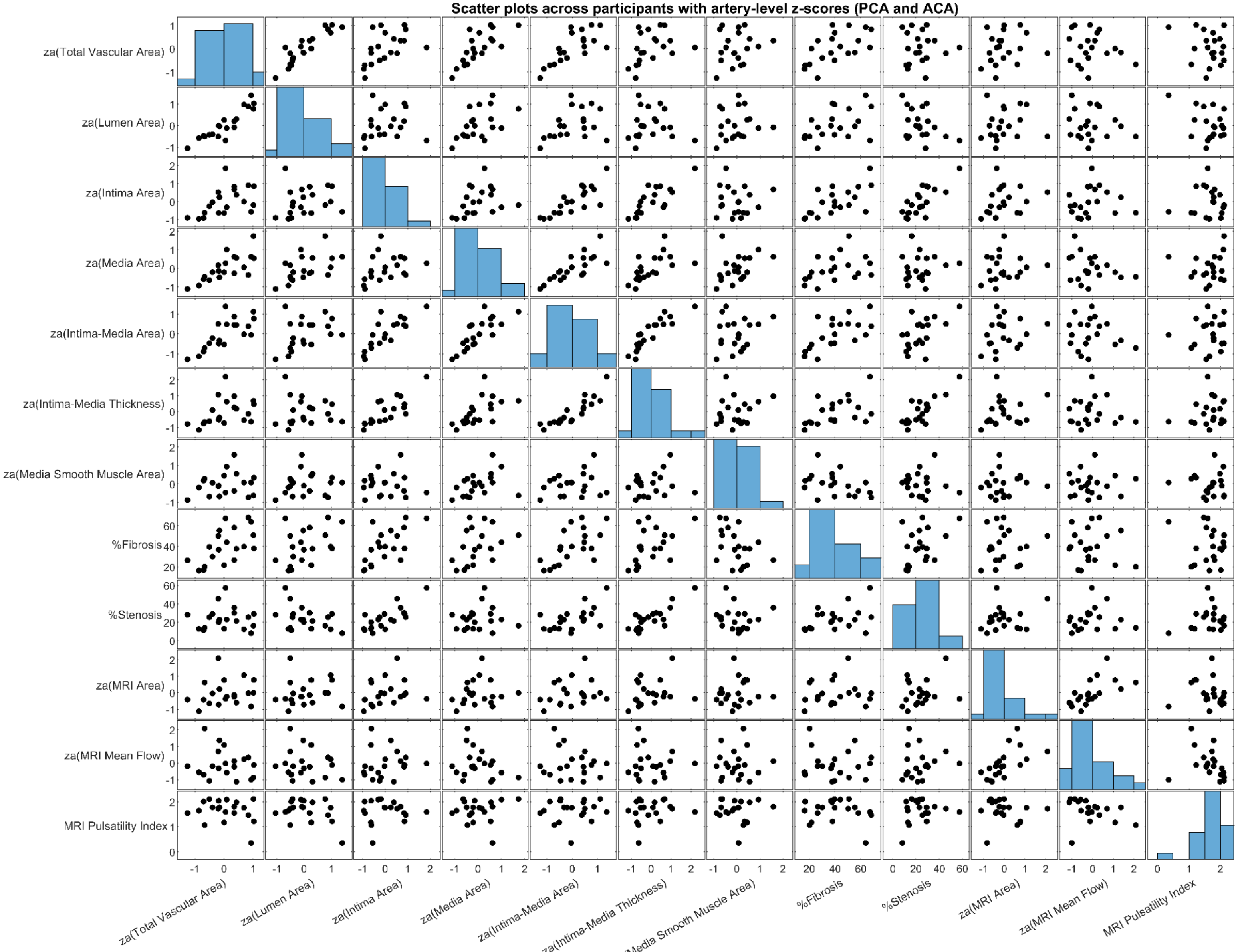
Scatter plots across participants with artery-level z-scores (PCA and ACA)
za(Total Vascular Area)
za(Lumen Area)
za(Intima Area)
za(Media Area)
za(Intima-Media Area)
za(Intima-Media Thickness)
za(Media Smooth Muscle Area)
%Fibrosis
%Stenosis
za(MRI Area)
za(MRI Mean Flow)
MRI Pulsatility Index

Supplemental Figure 10: Boxplots of cerebral artery morphology measures vs. brain pathology outcomes

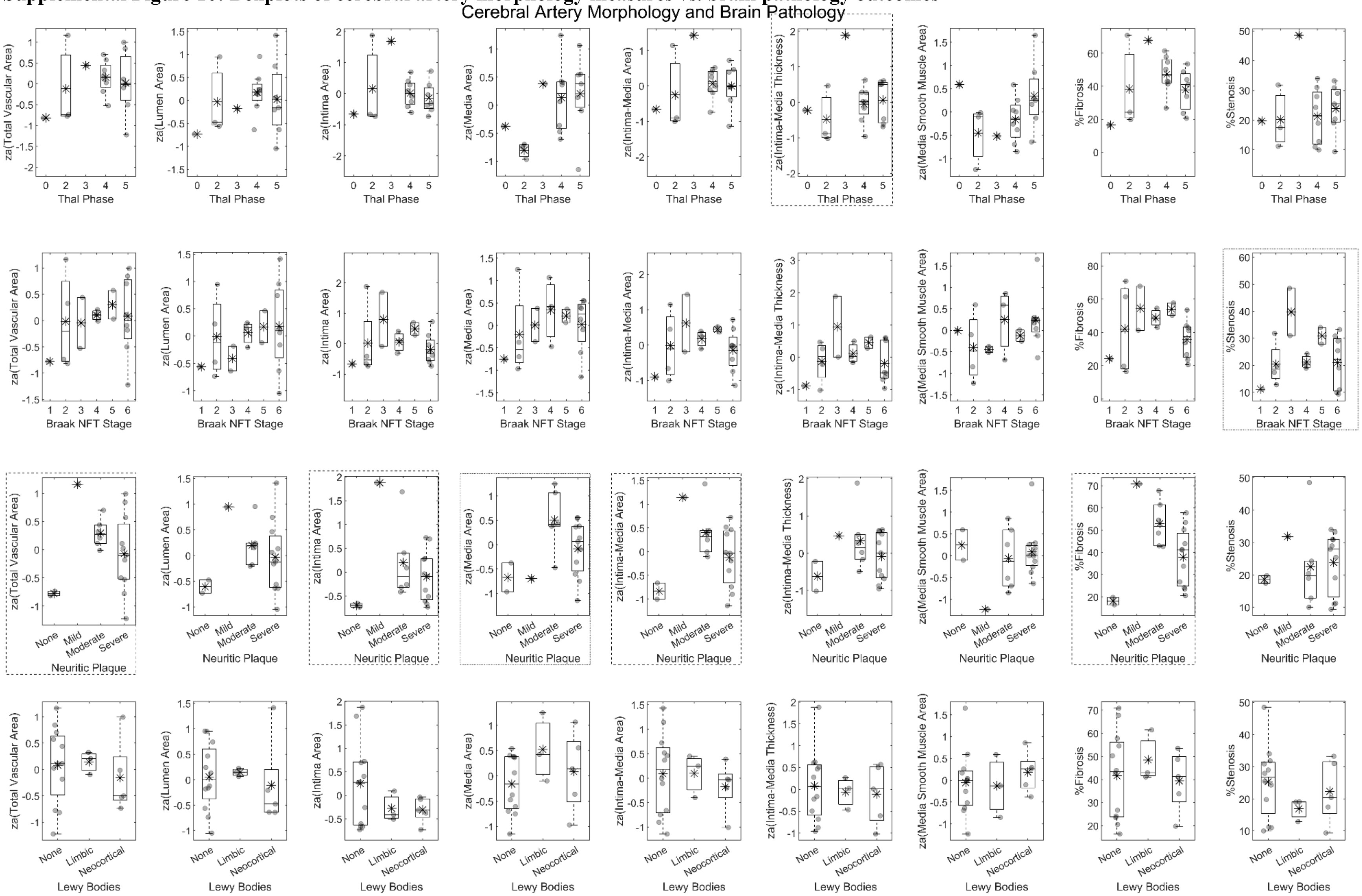

Plots enclosed with dashed lines represent p<0.05. Plots enclosed with dotted lines represent 0.05≤p≤0.1.

Supplemental Figure 11: Boxplots of cerebral artery morphology measures vs. TDP-43 brain pathology

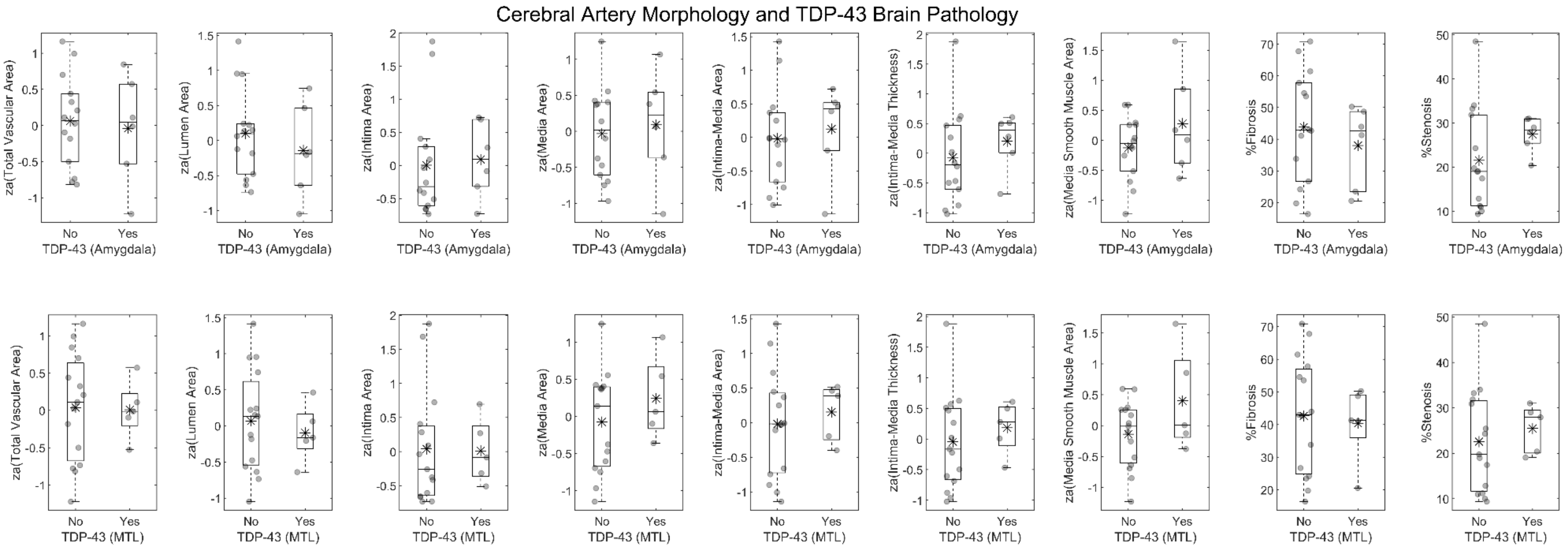

No arterial brain pathology measures were significantly associated with TDP-43 brain pathology.

Supplemental Figure 12: Boxplots of cerebral artery morphology measures vs. cerebrovascular pathology

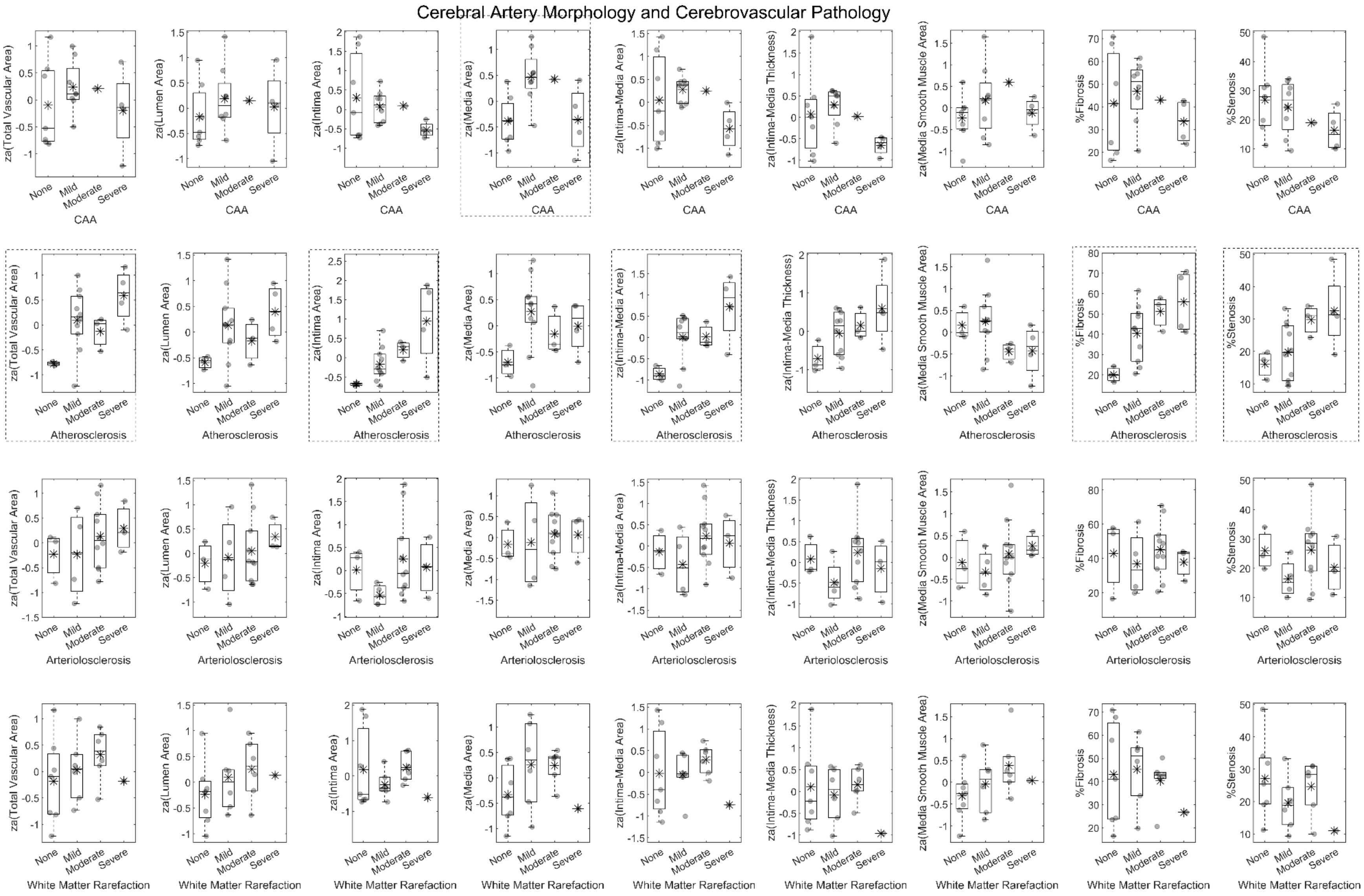

Plots enclosed with dashed lines represent $p<0.05$. Plots enclosed with dotted lines represent 0.05
